# Dehiscence, infection, seroma, haematoma (DISH): development and validation of a new classification of surgical site outcomes

**DOI:** 10.1093/bjs/znag112

**Published:** 2026-08-21

**Authors:** Giles Bond-Smith, Marja A Boermeester, David J Leaper, Philip L Russo, Antonia F Chen, the SSO Working Group Authors, Walter Danker III, Najmuddin Gunja, Anastasia Ermakova, Nikolaos Takatzoglou, Cindy Tong, Alex Pashley, Jessica Albutt, Alexandra Yoe, the SSO Expert Panel, Thomas Aubert, Toms Augustin, Emre Balik, Tomasz Banasiewicz, Jayme S Bennetts, Mujahid Bukhari, Marco Catarci, Mirian de Freitas Dal Ben Corradi, Eva Deerenberg, Christian Eckmann, Darío E Garín, Jignesh A Gandhi, Bijie Hu, HyungJin Kim, Hayato Kurihara, Suk-Hwan Lee, Varut Lohsiriwat, Christian Macutkiewicz, Najjia Mahmoud, Keita Morikane, Norikatsu Miyoshi, Manuel Novajas, Federico Nuñez, Ibrahim Obeid, Caroline E Reinke, Christine H Rohde, Ines Rubio-Perez, Esther A Saguil, Mauro J Salles, Angela Tecson, Jason Wellen, Yinmo Yang, the SSO Validation Study Panel, Yalda Afshar, Nitin Agarwal, Derek Amanatullah, Yaniss Belaroussi, Elias Castel, Alejandro Bolio Cerdán, Chong-Bum Chang, Patrick Charlebois, Paul Deramo, Federico Gorganchian, Shiki Fujino, Raj Gajbhiye, Zoilo Madrazo Gonzalez, Peter Graves, Dariusz Grzelecki, Chang Guohao, Dudy Arman Hanafy, Jonathan Hong, Susan Khalil, Bob Kiaii, Navamol Lekskul, Kil Yeon Lee, Shih-Hsuan Mao, Youn Young Park, Ji Won Park, Bhaveshkumar Patel, Luis Fernando Perin, Carlos Porras, Thiers Soares Raymundo, Christopher Reid, Rosa Jimenez-Rodriguez, Pablo Sanz-Ruiz, Nana Sarpong, Sharona Czerniak, Shahul Hameed Mohamed Siraj, Maureen Spencer, Santosh Thorat, Jonathan Torres-Castro, Konstantinos Toutouzas, Thomas Tsai, Francesco Venneri, Padmapriya Vivek, Mohamed Zayed

**Affiliations:** Department of Emergency General Surgery, Oxford University Hospitals NHS Foundation Trust, Oxford, UK; Department of Surgery, Amsterdam UMC, University of Amsterdam, Amsterdam, The Netherlands; Amsterdam Gastroenterology Endocrinology Metabolism, Amsterdam UMC, University of Amsterdam, Amsterdam, The Netherlands; Department of Surgery, University of Newcastle, Newcastle, UK; Department of Clinical Sciences, University of Huddersfield, Huddersfield, UK; Monash Nursing and Midwifery, Monash University, Melbourne, VIC, Australia; Cabrini Research, Cabrini Health, Melbourne, VIC, Australia; School of Nursing and Health, Avondale University, Lake Macquarie, NSW, Australia; Department of Orthopaedic Surgery, University of Texas Southwestern, Dallas, Texas, USA

## Abstract

**Background:**

Surgical site outcome (SSO) reporting lacks details of management, focuses on infection, and overlooks dehiscence, seroma, and haematoma. An international, consensus-driven classification system applicable to a diverse range of surgical incisions and outcomes was developed to address this gap and standardize the reporting of SSOs.

**Method:**

Concept statements based on a targeted literature review were rated by pan-specialty global experts during a four-round modified Delphi study to create a comprehensive classification of SSOs. Reliability and language validation focused on inter-rater agreement and intra-rater reproducibility by experts applying the classification to case vignettes, created using generative artificial intelligence sampling. Cross-domain agreement was evaluated using a patient-level, double entry, interclass correlation coefficient (DE-ICC).

**Results:**

Consensus was achieved on definitions of dehiscence, inflammation/infection, seroma, and haematoma (‘DISH’) within a new classification, comprising five objectifiable grades of clinical intervention (0–4) and four grades of clinical presentation (a–d) for each domain. The DISH ‘score’ presents all domains collectively, for example D1a I2b S0 H0; a snapshot of the management and presentation of an incision. Mean patient-level inter-rater DE-ICC was 0.93 (95% c.i. 0.91 to 0.96; median 1.00; very high concordance). Intra-rater DE-ICC was similarly high (mean 0.95; 95% c.i. 0.93 to 0.96; median 1.00).

**Conclusion:**

The proposed cross-specialty, global DISH Classification facilitates more comprehensive surveillance and audit of SSOs in clinical practice and research, by standardizing reporting of all relevant grades of clinical presentation and management of SSOs. Very high concordance when applied to case vignettes gives credibility to clinical validation and adoption.

## Teaser

An international panel of experts reached consensus on a universal classification for surgical site outcomes comprising dehiscence, infection, seroma, and haematoma, collectively referred to as ‘The DISH Classification System’. The proposed classification facilitates more comprehensive surveillance and audit of surgical site outcomes in clinical practice and research by standardizing reporting of their clinical presentation and management.

## Introduction

Surgical site outcomes (SSOs) are underreported, lack precise information on their clinical management, and focus on surgical site infections (SSIs) while overlooking dehiscence, seroma, and haematoma. Existing definitions and classifications for SSOs are limited.

The definitions and gradings advocated by the United States Centers for Disease Control and Prevention (CDC) for SSIs are the most used^1,2^. However, they focus solely on SSIs, failing to capture the burden of other SSOs including dehiscence, seroma, and haematoma, which are also costly to hospitals and patients^3^. In addition, the CDC classification system relies heavily on subjective clinical judgement, which can be variable across institutions, leading to ambiguity in interpretation^1,2^. This could contribute to incorrect diagnoses and suboptimal wound management, which can have negative impacts on patient outcomes such as impaired wound healing, increased risk of adverse events, and reduced quality of life^4^. The CDC classification system is also merely a descriptive classification of clinical presentation, that does not account for subsequent related management. Standardized tracking of the clinical management of wounds would facilitate comparisons of clinical practice and benchmarking of outcomes between institutions, which could be used to inform quality improvement^5^. These data may also support research or audit across surgical specialties.

All SSOs can be barriers to optimal healing of surgical incisions and can occur independently or interdependently. Their impact can also vary depending on the surgical specialty and anatomical site. For example, dehiscence can occur without a prior SSI, and its severity increases with the depth of tissue involvement^6^. Dehiscence of an abdominal or sternotomy wound, or after hip or knee prosthetic surgery, can be life-threatening and may require prolonged interventional management. Exposed dehisced wounds are prone to secondary infection.

Seromas and haematomas can also present substantial challenges, depending on surgical specialty. Postoperative seromas are common, particularly after mastectomy^7^, and may require repeated aspiration, interventional imaging, or even open management, all of which may delay adjuvant chemotherapy or radiotherapy, potentially impacting survival outcomes. Haematomas, which can range from minor bruising to large collections in soft tissues or visceral cavities, are at risk of becoming infected, leading to abscess formation and sepsis. They may also require emergency surgical intervention to evacuate the haematoma and control bleeding.

A comprehensive classification system that defines all postoperative incisional outcomes (dehiscence, infection, seroma, and haematoma), and enables standardization of terms used in clinical practice, irrespective of speciality, procedure or setting, could form a framework to improve SSO reporting and ultimately their management and outcome. It could also be useful as an interventional scale for clinical research, audits and budget planning for all surgical specialties, as well as reducing the variability in reporting.

This study aims to establish and validate an international, consensus-driven classification system applicable to a diverse range of surgical incisions to standardize the reporting of SSOs.

## Methods

**Figure 1:**
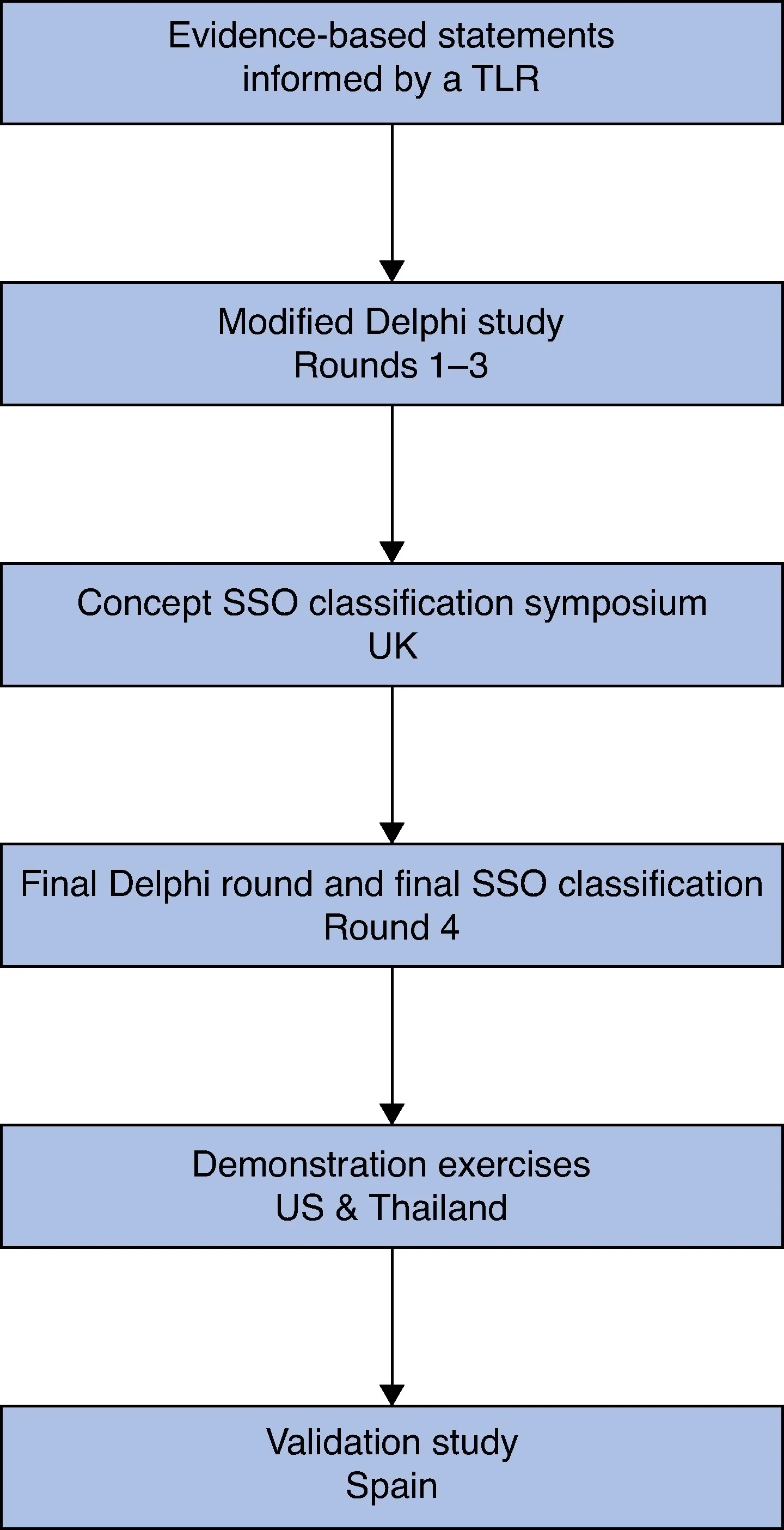
**Overview of studies** SSO, surgical site outcome; TLR, targeted literature review.

The stepwise creation and validation of a new SSO classification is outlined in *Figure 1*.

### Evidence-based statements

A TLR was undertaken to identify key themes and gaps in relation to dehiscence, inflammation/infection, seroma, and haematoma. Data from structured and ad-hoc searches of MEDLINE and Embase via Ovid SP, MEDLINE via PubMed, and Google Scholar (Supplementary Information: *Appendix S1*) were used to develop definition statements for the Delphi study. The TLR was not intended as a systematic review and was scoped to generate concept statements for Delphi discussion rather than provide a definitive evidence synthesis.

### Modified Delphi study

Concept statements based on the findings from the TLR were rated by a 32-member Expert Panel from 22 countries spanning multiple specialties. This took place over three rounds of anonymous voting, until a comprehensive classification of SSOs was established. Further details of the methodology can be found in Supplementary Information: *Appendices S2* and *S3*.

### Concept SSO classification and debate amongst international experts

An in-person symposium brought together all panellists alongside other international experts to debate and critique the newly proposed classification system.

### Final Delphi round and final SSO classification

Feedback from the symposium was used to inform the final version of the classification, which was agreed on following a fourth and final round of anonymous voting from the Expert Panel.

### Validation study

A follow-up study to validate the reliability and language of the novel classification system was undertaken at a second in-person symposium. The design of this validation study was informed by preliminary demonstration exercises performed in the USA and Thailand. These exercises were exploratory versus the validation process described below, which was conducted according to a prespecified protocol (Supplementary Information: *Appendix S4*).

Validation was assessed via inter-rater agreement (the concordance between participant’s scores for the same case) and intra-rater reproducibility (the concordance of a participant’s scoring of the same set of cases in reverse order). Nineteen patient case vignettes were created using a rule-based generative artificial intelligence (Gen-AI) pipeline from a constrained ‘universe’ of allowable vignettes spanning eight ‘DISH’ (Dehiscence, Infection, Seroma, and Haematoma) scoring domains (four complication domains, each with presentation, P and intervention, I). Using a coverage-driven approach, vignettes were selected using a stratified sampling algorithm that ensured full representation of all allowed within-domain (P, I) pairs; the set also included one all-zero vignette with uneventful healing. Vignettes were structurally capped at a maximum of two active domains to ensure clinical plausibility while limiting the number of cases and minimizing rater burden. Ultimately, 19 patient case notes were generated from the patient case vignettes using Gen-AI and were independently reviewed for face-validity ahead of the study.

A panel of international surgeons and infection control specialists representing a broad range of specialties was convened. Participants received comprehensive training on the DISH scale before completing in-person scoring of the patient case vignettes under controlled conditions across two sessions over two days.

Cross-domain agreement was evaluated using a patient-level double entry intra-class correlation coefficient (DE-ICC), which quantifies agreement on the full eight-component DISH score (vector comprising four domains × presentation/intervention). DE-ICC provides a single summary of concordance across the full DISH scoring profile for each patient, capturing whole-profile agreement (overall and cross-domain). DE-ICC directly summarizes whole-case agreement across all eight DISH components, aligning with the study’s primary objective of cross-domain reliability. Domain-level agreement statistics were reported to support identification of which DISH components drive disagreement and to understand where refinements to the classification or training materials may be required.

Domain-level inter-rater agreement was assessed using Kendall’s coefficient of concordance (*W*). Quadratic-weighted Gwet’s AC2 with 95% confidence intervals was computed as a supplementary ordinal agreement metric less sensitive to class imbalance. Both mean accuracy (overall exact agreement) and balanced mean accuracy (macro-averaged across severity levels) were calculated.

Intra-rater reproducibility was assessed using the DE-ICC framework on repeated ratings in the second validation session of the same patient notes, with the order reversed to reduce recall bias from the prior session.

## Results

### Targeted literature review

*Table 1* explores the limitations of common systems compared to the new DISH Classification System. The CDC, Southampton Wound Assessment Scale, and ASEPSIS systems were selected as comparators as they were the most widely used and/or cited general SSO-related systems identified in the TLR, making them the most relevant comparators for illustrating gaps in current practice.

**Table 1:** Illustrative comparison of different surgical outcome and infection classification systems

Domain	Centers for Disease Control and Prevention (CDC)^1^	Southampton Wound Assessment Scale^8^	ASEPSIS Wound Score^9^	Proposed DISH Classification
Applicability across surgical specialties	Yes	No	Partially	Yes
Includes clinical presentation	Yes	Yes	Yes	Yes
Includes clinical intervention/management	Partially	No	Partially	Yes
Generates an overview score	No	Yes	Yes	Yes
Can be interpreted by all members of the surgical team	Partially	Partially	No	Yes
Defines and classifies dehiscence (D)	No	No	No	Yes
Defines and classifies infection (I)	Yes	Partially	Partially	Yes
Defines and classifies seroma (S)	No	No	No	Yes
Defines and classifies haematoma (H)	No	No	No	Yes
Domains (D, I, S, H) graded independently	No	No	No	Yes

The review also identified variable definitions for dehiscence, infection, seroma, and haematoma, demonstrating the need for standardized definitions to ensure consistent reporting of surgical outcomes. Detailed TLR results are in Supplementary Information: *Appendix S5*.

### Modified Delphi study and finalization of classification

Although consensus was reached on a new classification of SSOs following Round Three of the modified Delphi study, the in-person symposium prompted further refinement (see Supplementary Information: *Appendices S6* and *S7*). A fourth round was initiated, with statements drafted based on those from the previous rounds and symposium feedback. This resulted in the dissociation and independent scoring of clinical intervention and clinical presentation, with five options grading the intervention received (0–4) and four grading the clinical presentation observed (a–d).

**Figure 2:**
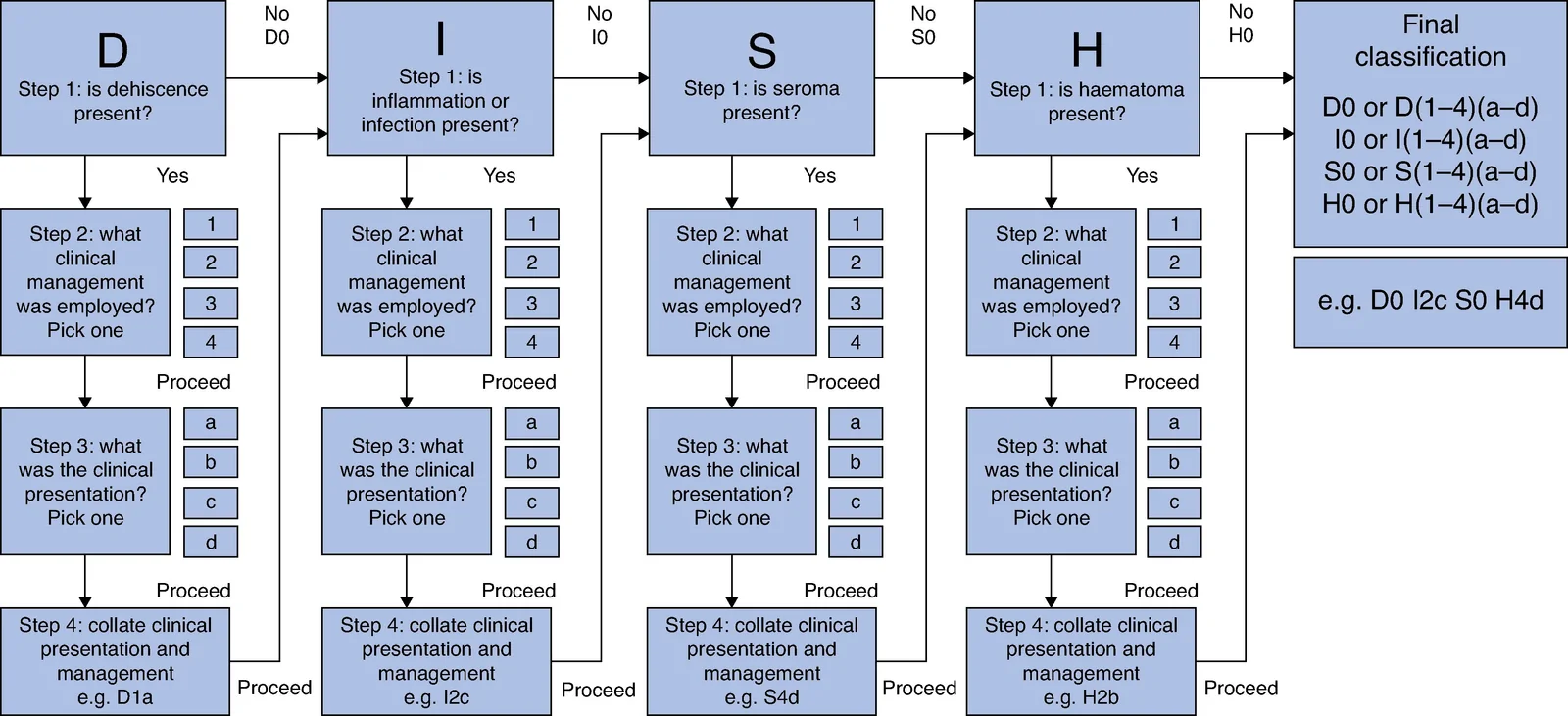
**How to use the DISH classification system** D, dehiscence; I, inflammation/infection; S, seroma; H, haematoma.

The proposed final universal classification, the DISH Classification System for SSOs, is described in *Table 2* (clinical intervention/management) and *Table 3* (clinical presentation). The final DISH ‘score’ presents a snapshot of the management received and the presentation of an incision, for example D1a I2b S0 H0 (*Fig. 2*).

**Table 2:** Proposed DISH classification system: clinical management

	Dehiscence	Inflammation/Infection	Seroma	Haematoma
0	None	None	None	None
1	Patient self-monitoring	Patient self-monitoring	Patient self-monitoring	Patient self-monitoring
2	Local treatment, with or without local anaesthesia	Healthcare professional monitoring; closed incision management; antibiotics optional	Healthcare professional monitoring with or without radiological imaging	Healthcare professional monitoring
3	Surgical reintervention with general or regional anaesthesia, with simple incisional reapproximation (closure)	Surgical reintervention with local anaesthesia, or percutaneous drainage; antibiotics optional	Local treatment with local anaesthesia, or percutaneous drainage	Local treatment with local anaesthesia, or percutaneous drainage, or embolization
4	Surgical reintervention with general or regional anaesthesia, with complex reapproximation (reconstruction) or leaving wound open (no reapproximation or reconstruction)	Surgical reintervention, with general or regional anaesthesia	Surgical reintervention, with general or regional anaesthesia	Surgical reintervention or radiological intervention with general or regional anaesthesia

**Table 3:** Proposed DISH classification system: clinical presentation

	Dehiscence	Inflammation/Infection	Seroma	Haematoma
a	Partial separation of the incision edges with no to minimal fluid discharge	One or more of the following clinical signs of inflammation related to the incision: erythema or other visible skin colour change, swelling, heat, pain, or serous exudate	Presence of localized fluid collection related to the surgical incision with no to minimal discomfort or pressure in the affected area	Bruising related to the incision, with or without swelling, but not causing substantial symptoms to the patient
b	Substantial separation of the incision edges, without disrupting fascia or the deep tissue	Two or more of the following clinical signs: erythema or other visible skin colour change, swelling, heat, pain, non-serous exudate; plus one or more of the following: elevated C-reactive protein, increased serum white blood cell count, temperature over 38°C (100.4°F), positive wound or blood culture, gas in tissues	Large fluid collection causing moderate discomfort or pressure in the affected area	Enlarging haematoma causing swelling and pain
c	Extensive separation of the incision edges, involving deep tissue layers	Severe infection related to the incision with systemic signs of sepsis or mono-organ failure	Large fluid collection causing significant clinical signs or symptoms	Significant haematoma causing substantial swelling, pain, and compromising nearby organs or structures
d	Most severe incision dehiscence of all surgical tissue layers with or without organ exposure	Septic shock; Sepsis related multi-organ failure	Chronic walled-off or large fluid collection causing life-threatening symptoms	Significant haematoma, with active bleeding

### Validation study

A second symposium took place in Madrid, Spain, 19–20 February 2026. A total of 64 surgeons and infection control specialists participated, of whom 60 provided full responses in both rating sessions. An example validation case note with step-by-step DISH scoring guidance is provided in Supplementary Information: *Appendix S8*.

Domain-level inter-rater agreement was consistently high. Mean Kendall’s *W* was 0.96 (range 0.93–0.97). Gwet’s AC2 averaged 0.96 (range 0.94–0.99), with all domain-specific 95% confidence intervals lower bounds above 0.85, indicating ‘excellent’ agreement. Mean accuracy across domains was 93% (range 87–97%), and balanced mean accuracy was 87% (range 77–95%), suggesting accuracy was not driven solely by the most common severity levels. Mean patient-level inter-rater DE-ICC was 0.93 (95% c.i. 0.91 to 0.96; median 1.00), indicating very high concordance in raters’ 8-domain scoring profiles for the same patient. Intra-rater DE-ICC was similarly high (mean 0.95; 95% c.i. 0.93 to 0.96; median 1.00).

Sensitivity analysis excluding zero-valued observations within each domain and scoring dimension yielded lower mean accuracy across all domains, most notably for dehiscence (intervention; 78%), and infection and seroma (presentation: 77% and 75% respectively); discrepancies in these domains were similarly noted during the symposium through discussion and following crude analysis of the results. Analysis of feedback was used to make minor edits to the classification’s language, which are reflected in the classification shown in *Tables 2* and *3*. Full results are detailed in the Supplementary Information: *Appendix S9*.

## Discussion

This study addresses the need for a universal classification system spanning the wide range of SSOs, achieved through international consensus and validation. The comprehensive, cross-specialty global DISH Classification is an evidence-based and consensus-driven definition and classification for SSOs, comprising dehiscence, inflammation/infection, seroma, and haematoma. The system encompasses definitions for each domain, grading severity for clinical intervention and management, and grading severity for clinical presentation. Given differences between specialties, no fixed timepoint to collect DISH is prescribed, though day of discharge and at usual fixed follow-up points are advised. For surgery involving implants, it is recommended that the follow-up period over which DISH is recorded should be at least one year, given the potential for delayed onset of complications. However, this may vary based on local postoperative surveillance protocols. The DISH classification had a very high mean patient-level inter-rater DE-ICC of 0.93 (95% c.i. 0.91 to 0.96; median 1.00; very high concordance) and a similarly high mean intra-rater DE-ICC of 0.95 (95% c.i. 0.93 to 0.96; median 1.00). DISH has gained cross-specialty and cross-country acceptance as a clinically powerful surveillance, registration, research and audit tool, still in need for further widespread validation in clinical practice.

Given the absence of standardized definitions for SSOs in current practice, specifically for dehiscence, seroma, and haematoma, there is a need for this novel classification system, which could improve SSO reporting across all surgical specialities. There are currently few classification systems available that allow clinicians to report, classify, and grade SSOs they observe. The CDC classification for SSIs, the Southampton Wound Assessment Scale, and the ASEPSIS Wound Score system all fail to fully encompass SSOs other than infection, use definitions with limited practical and clinical relevance, lack information about management, and have limited utility for non-surgeons^1,8,9^. With the exception of the CDC classification, they also lack applicability to different surgical specialities^1,8,9^. It is not possible to compare these classifications directly, as the function and intention of each is different. *Table 1* highlights the limitations of existing classifications and demonstrates why a new classification system was needed to grade SSOs.

The widespread applicability of the DISH Classification System is its focus on creating a ‘common language’ for recognizing SSOs, including their specificity and severity. It allows for the accurate identification of the four domains (dehiscence, infection/inflammation, seroma, and haematoma), and their true incidence and severity, thus guiding targeted and effective prevention. Improving the accuracy of identification, reporting, and surveillance of SSOs will support the development and implementation of universal guidelines and care bundles across different surgical specialties. Strong inter- and intra-rater reliability in the validation study is supportive of the DISH score being universally applicable in clinical practice. However, the DISH Classification System is viewed as a starting framework for future work; clinical validation and prospective studies are needed, and it is anticipated that the DISH Classification System will evolve over time. Pilot exercises across different surgical specialities involving healthcare providers in many regions are planned or underway, that aim to determine the feasibility, acceptability, and validity of the DISH Classification System in real-world settings.

A systematic approach was used to gather expert consensus, and the anonymity of the process encouraged transparency from participants in this study. The study engaged an Expert Panel of 32 individuals from 22 countries, who represented a diverse group of surgeons across various surgical specialities and infection control specialists, although not all viewpoints may have been represented. These factors strengthen the validity of the final definitions of each domain and support the universal applicability of the proposed DISH Classification System.

A modified Delphi method was adopted rather than the ‘pure’ Delphi method, which typically requires ongoing rounds until full consensus is achieved^10^, and represents a lower evidence level compared with systematic review and meta-analysis. However, feedback from the in-person symposia, validation study, and peer review have further strengthened the DISH Classification System, helping to ensure that it is applicable to all patients and useful to the healthcare community.

Although the results of the validation exercise are encouraging, this is not a substitute for clinical validation. Despite efforts to standardize the grading criteria, some degree of subjectivity remains inherent to any ordinal clinical grading system. Patient involvement was not incorporated at any stage of the study, representing a further limitation that will be addressed as a priority in the planned real-world pilot phase. AI-generated patient case vignettes represent an ‘ideal’ case, whereas real-world case notes are likely to be more ambiguous. AI-generated vignettes allowed standardized control of case content across all scenarios and avoided the consent, privacy, and logistical complexity of using real patient data, but future studies will apply the classification to real patient data. Fringe cases were excluded, thereby focusing on the most ‘likely’ DISH cases healthcare professionals see. Similarly, not all potential specialties could be captured in the generated case notes, which may reduce external validity. Additionally, some specialties were underrepresented on the Expert and Validation Panels; increasing the representation of these specialties is a target of planned real-world pilot studies. The validation exercise was also limited to clinicians only, representing an important but nonetheless incomplete cross-section of expected users. Evaluating use by the wider clinical team, in real-world settings, is an objective of planned real-world studies. Additional analyses, such as role, specialty, native English fluency, setting, and learning curve need to be considered.

The proposed cross-specialty, global DISH Classification facilitates comprehensive surveillance and audit of SSOs in clinical practice and research, by standardizing reporting of all relevant grades of clinical presentation and clearly objectifiable management of SSOs. Very high concordance when applied to case vignettes gives credibility to clinical adoption and subsequent validation.

## Collaborators

**SSO Working Group Authors:** Walter Danker III, PhD; Najmuddin Gunja, PhD; Anastasia Ermakova, PharmD; Nikolaos Takatzoglou, MSc; Cindy Tong, MSc; Alex Pashley, MChem; Jessica Albutt, MSc; Alexandra Yoe, MSc.

**Affiliations of the SSO Working Group Authors:** Johnson & Johnson, Raritan, USA (Danker, Ermakova, Tong); Johnson & Johnson, Ontario, Canada (Gunja); Johnson & Johnson, Athens, Greece (Takatzoglou); Costello Medical, Cambridge, UK (Pashley); Costello Medical, London, UK (Yoe [at the time of study], Albutt).

## Supplementary Material

znag112_Supplementary_Data

## Data Availability

Deidentified individual participant data from the validation will not be made available after publication by default, but reasonable requests to access these data will be considered and should be directed to the corresponding author along with a research proposal. Proposals will be reviewed by the publication authors, and data will be shared upon approval.
