# Supplementary material for: Dehiscence, infection, seroma, haematoma (DISH): development and validation of a new classification of surgical site outcomes
Source: Br J Surg. 2026 Aug 21;113(9):znag112. doi: 10.1093/bjs/znag112 (PMC13584926; doi:10.1093/bjs/znag112)
Supplement: Supplementary file 1 [file znag112_supplementary_data.docx]

**Supplementary Material for: Dehiscence, Infection, Seroma, Haematoma (DISH): Development and Validation of a New Classification of Surgical Site Outcomes**

Giles Bond-Smith, FRCS^1^; Marja A. Boermeester, MD^2,3^; David J. Leaper, DSc^4,5^; Philip L. Russo, PhD^6,7,8^; Antonia F. Chen, MD/MBA^9^; and the SSO Working Group Authors; on behalf of the SSO Expert Panel and the SSO Validation Study Panel

^1^Oxford University Hospitals NHS Foundation Trust, Oxford, United Kingdom

^2^Department of Surgery, Amsterdam UMC, University of Amsterdam, Amsterdam, The Netherlands

^3^Amsterdam Gastroenterology Endocrinology Metabolism, Amsterdam UMC, University of Amsterdam, Amsterdam, The Netherlands

^4^University of Newcastle, Newcastle, UK

^5^University of Huddersfield, Huddersfield, UK

^6^Monash Nursing and Midwifery, Monash University, Australia

^7^Cabrini Research, Cabrini Health, Australia

^8^Avondale University, Australia

^9^Department of Orthopaedic Surgery, University of Texas Southwestern, Dallas, Texas, USA

**Corresponding author.** Mr. Giles Bond-Smith, Clinical Director Surgery, Oxford University Hospitals NHS Foundation Trust, Headington, Oxford, OX3 9DU ([giles.bond-smith@ouh.nhs.uk](mailto:giles.bond-smith@ouh.nhs.uk))

ORCID ID 0000-0001-7385-7521

**Supplementary Materials – Index**

Supplementary Appendixes

[Appendix 1: Targeted Literature Review (TLR) Search Protocol	*page* 4](#_Toc236660214)

[1.1 Objective	*page* 4](#_Toc236660215)

[1.2 Approach	*page* 4](#_Toc236660216)

[1.2.1 Literature Sources	*page* 4](#_Toc236660217)

[1.2.2 Search Strategy	*page* 4](#_Toc236660218)

[1.2.3 Review Process	*page* 5](#_Toc236660219)

[1.2.4 Quality Assessment	*page* 5](#_Toc236660220)

[Appendix 2: Modified Delphi Study Methodology	*page* 6](#_Toc236660221)

[2.1 Modified Delphi Consensus Design	*page* 6](#_Toc236660222)

[2.1.1 Overview	*page* 6](#_Toc236660223)

[2.1.2 Rounds One to Four	*page* 6](#_Toc236660224)

[2.1.3 Post-Round Three Symposium	*page* 6](#_Toc236660225)

[2.2 Participants in the Modified Delphi Study	*page* 6](#_Toc236660226)

[2.3 Expert Panel Member Selection	*page* 7](#_Toc236660227)

[2.3.1 Expert Panel Member Selection Criteria	*page* 8](#_Toc236660228)

[2.3.2 Criteria Application Process	*page* 9](#_Toc236660229)

[2.3 References	*page* 10](#_Toc236660230)

[Appendix 3: ACCORD Checklist	*page* 11](#_Toc236660231)

[Appendix 4: Validation Study Protocol	*page* 14](#_Toc236660232)

[4.1 Introduction	*page* 14](#_Toc236660233)

[4.1.1 Background	*page* 14](#_Toc236660234)

[4.1.2 DISH Classification System	*page* 14](#_Toc236660235)

[4.1.3 Validation Study	*page* 14](#_Toc236660236)

[4.2 Key Objectives	*page* 15](#_Toc236660237)

[4.3 Methods	*page* 15](#_Toc236660238)

[4.3.1 Study Design	*page* 15](#_Toc236660239)

[4.3.2 Validation Methodology	*page* 16](#_Toc236660240)

[4.3.3 Generation of Simulated Case Notes	*page* 19](#_Toc236660241)

[4.3.4 Analyses	*page* 21](#_Toc236660242)

[4.3.5 Ethics	*page* 22](#_Toc236660243)

[4.3.6 References	*page* 23](#_Toc236660244)

[Appendix 5: Targeted Literature Review Results	*page* 24](#_Toc236660245)

[5.1 Overview	*page* 24](#_Toc236660246)

[5.2 Dehiscence: Current Definitions and Reporting Guidelines	*page* 24](#_Toc236660247)

[5.3 Infection: Current Definitions and Reporting Guidelines	*page* 25](#_Toc236660248)

[5.4 Seroma: Current Definitions and Reporting Guideline	*page* 27](#_Toc236660249)

[5.5 Haematoma: Current Definitions and Reporting Guidelines	*page* 28](#_Toc236660250)

[5.6 Current Barriers to Reporting Surgical Outcomes and HCP Perceptions of SSIs	*page* 30](#_Toc236660251)

[5.7 Overview of Existing Classification Systems	*page* 31](#_Toc236660252)

[Appendix 6: Supplementary Results for the Modified Delphi Study	*page* 34](#_Toc236660253)

[6.1 Rounds One to Three	*page* 34](#_Toc236660254)

[6.2 Post-Round Three Symposium	*page* 34](#_Toc236660255)

[6.3 Detailed Delphi Panel Results	*page* 34](#_Toc236660256)

[6.3.1 Delphi Panel Results Round One	*page* 34](#_Toc236660257)

[6.3.2 Delphi Results Round Two	*page* 37](#_Toc236660258)

[6.3.3 Delphi Results Round Three	*page* 38](#_Toc236660259)

[6.3.4 Delphi Results Round Four	*page* 38](#_Toc236660260)

[Appendix 7: Post-Round Three Symposium	*page* 41](#_Toc236660261)

[7.1 Critique of the Proposed Classification	*page* 41](#_Toc236660262)

[7.2 Pilot exercise of the proposed classification after Round Three (independent of/prior to the formal validation exercise)	*page* 41](#_Toc236660263)

[Appendix 8: Validation Case Worked Example	*page* 43](#_Toc236660264)

[8.1 Example Case Note	*page* 43](#_Toc236660265)

[8.2 DISH Scoring Process	*page* 43](#_Toc236660266)

[8.2.1 Dehiscence	*page* 43](#_Toc236660267)

[8.2.2 Inflammation/Infection	*page* 43](#_Toc236660268)

[8.2.3 Seroma	*page* 44](#_Toc236660269)

[8.2.4 Haematoma	*page* 44](#_Toc236660270)

[8.2.5 Overall DISH Score	*page* 45](#_Toc236660271)

[Appendix 9: Supplementary Validation Results of SSO rating by DISH	*page* 46](#_Toc236660272)

Appendix 1: Targeted Literature Review (TLR) Search Protocol

1.1 Objective

The objective of the TLR is to identify key themes, as reported in the scientific literature, in relation to the universal classification. The literature identified is anticipated to consider current barriers to reporting of surgical site outcomes, as well as the clinical and economic burden associated with diagnosis of each component, providing the rationale to develop and implement the universal classification. Furthermore, searches will look to identify current definitions and reporting guidelines for each component of the endpoint to support development of statements for the first round of the Delphi consensus study.

1.2 Approach

1.2.1 Literature Sources

We will search the following electronic databases to identify relevant published literature:

- MEDLINE, including MEDLINE In-Process, MEDLINE Daily and MEDLINE Epub Ahead of Print (to be searched via the Ovid SP platform and PubMed)
- Embase (to be searched via the Ovid SP platform)

Search engines such as Google Scholar will also be employed, as well as reviews of reference lists from relevant articles. To support identification of guidelines, which are often not identified via Embase and MEDLINE, searches of [GuidelineCentral](https://www.guidelinecentral.com/guidelines/) and key health technology assessment (HTA)/national society bodies will be conducted where required.

1.2.2 Search Strategy

The searches will be conducted to find information on key themes in relation to the universal classification by combining keywords related to each endpoint and the areas of interest outlined below. No geographical limits will be applied. The following themes will serve as a starting point, with the potential to include additional, relevant topics as they are identified during the search:

- Healthcare professional perceptions of surgical site infection (SSI) and barriers to reporting of SSI to contextualise the need for a novel universal classification that moves away from the language of SSI, supported by any evidence on the benefits of a universal classification in healthcare outcome reporting
- An overview of each component of the proposed universal classification (dehiscence, infection, seroma, haematoma), including:
  - Any measures related to the quality and/or consistency of reporting, with a view to outlining current barriers to reporting
  - Current definitions of the outcomes, as outlined by guidelines and consensus documents, including any scoring systems or measures of severity currently in practice. This will provide background to the development of statements for Round One of the Delphi, whilst also providing an overview in the pre-reads of the current *status quo* for each component

Given the broad nature of the topics of interest and the targeted nature of the review, the searches will be conducted in a time-limited manner and tailored depending on the level of available evidence. For topics where there is substantial information, studies published more recently, and studies of a higher evidence level will be prioritised. When considering evidence level, preference will be given to systematic literature reviews and meta-analyses, followed, in order, by randomised controlled trials (RCTs), interventional non-RCTs, prospective observational studies, and retrospective database and observational analyses.

Initial searches will be conducted for each key theme and the first 50 results, ordered by relevance (PubMed, Google Scholar) and year of publication (Ovid SP; most recent first), will be reviewed. For key relevant articles, citations to other related publications will be investigated and the ‘similar articles’ feature within Google Scholar will be utilised to identify further relevant publications. Results will be assessed, and the strategy will be modified as needed. Different searches will be iterated through, adding less specific search terms if needed, or addressing specific gaps. Searches for each area will be time-limited and that, or the reasonable exhaustion of relevant literature, will determine the conclusion of searches in a particular area. A record will be kept of all searches that have been run.

1.2.3 Review Process

The review process will be as follows:

- Each title, or abstract/full-text article as needed, will be reviewed by a single reviewer. While formal eligibility criteria will not be used
- Throughout the review process, relevant articles will be documented, and top-line information will be captured by a single reviewer

1.2.4 Quality Assessment

No formal quality assessments will be conducted as part of the TLR.

Appendix 2: Modified Delphi Study Methodology

2.1 Modified Delphi Consensus Design

2.1.1 Overview

A modified Delphi study was conducted in accordance with the ACcurate COnsensus Reporting Document (ACCORD) guidelines (Appendix 3).^1^ This robust methodology is commonly used to generate expert consensus in healthcare through iterative survey rounds answered anonymously.^1, 2^ Three rounds of the Delphi study were completed prior to an in-person symposium, which brought together all Delphi contributors alongside other clinical experts to discuss and critique the proposed universal classification system. Outcomes from this symposium were used to inform and initiate a fourth round of the Delphi study. Following establishment of consensus, validation of the language and reliability of the classification among surgeons and infection control specialists was established at a second, in-person symposium.

The modified Delphi study was conducted via an online survey, including Likert scales, multiple choice and ranking, with free-text responses available for all statements. Voting was anonymous, with free-text responses enabling additional insight to be gathered. All statements in Rounds Two to Four were based on a four-point Likert Scale (strongly agree, agree, disagree, strongly disagree). The consensus threshold was defined as ≥75% agreement or disagreement. An exemption was granted by the Institutional Review Board at Oxford University Hospitals on the basis that the study constituted a pre-research activity and did not involve human subjects.

2.1.2 Rounds One to Four

The TLR results informed the statements for Round One. Statements that did not reach consensus were removed, revised or reissued in the subsequent round, informed by free-text responses (and via the symposium for Round Four) (Supplementary Figure 1). Panellists received a summary of anonymised feedback after each round, including the proportions of participants selecting each response option and the free-text responses.

2.1.3 Post-Round Three Symposium

The first symposium took place after Round Three to critique the proposed universal classification system and facilitate debate among experts in their respective fields.

2.2 Participants in the Modified Delphi Study

A Steering Committee of five experts (GBS, MAB, DJL, PLR, and AFC) was formed to guide the consensus process. The criteria for selecting the Steering Committee required that they were renowned professionals of national and/or international significance, with expertise in SSO epidemiology and demonstrable integrity.^3^ To avoid bias, neither the Steering Committee nor Sponsor actively participated in the consensus gathering process. The Expert Panel consisted of 32 participants from 22 countries, who provided expertise in various surgical disciplines, infectious diseases, and infection prevention and control (Supplementary Figure 1).

Panellists were recruited using pre-specified selection criteria and a sampling framework, ensuring appropriate content expertise, no relevant conflicts of interest and broad international representation.

**Supplementary Figure 1: Summary of the Delphi Process and Results**

**Footnotes:** ^a^Three statements were non-consensus generating. ^b^One Expert Panel member was excluded from the process prior to Round Three due to non-response in Round Two. ^c^Two Expert Panel members did not respond in Round 4. ^d^Some participants had multiple specialties.

**Abbreviations:** D: dehiscence; I: inflammation/infection; S: seroma; H: haematoma.

2.3 Expert Panel Member Selection

The composition of the Delphi Expert Panel members, and the criteria by which they are selected, is of critical importance. Expert Panel members will participate in the Delphi panel by providing their expertise through the completion of anonymous Delphi surveys and by participating in the Expert Meeting. The validity of the consensus statements developed by the Delphi panel is therefore dependent on Expert Panel members being content experts and without conflicts of interest.^4^

2.3.1 Expert Panel Member Selection Criteria

Selection of the Delphi Expert Panel members will be organised in consultation with the Steering Committee members. The Steering Committee will play an important role in helping identify the criteria for the panellist involvement. First and foremost, all Expert Panellists must meet the following criteria:

1. Be able to commit to the time required to participate in the Delphi panel
2. Understand the survey contents and provide constructive and critical feedback in English
3. Work in one of the priority surgical areas (colorectal, hepatobiliary, general, orthopaedic [specialising in total joint arthroplasty], cardiovascular, gynaecology, plastic, spine or thoracic) and/or work in wound care or infection management

Then, considering the global relevance and cross-specialist output of this Delphi panel, a unique sampling framework is required as depicted in Supplementary Figure 2. Thirty experts will be purposefully recruited from G20 countries across three regions (Asia, the Americas and Europe), with the target representation outlined in Supplementary Figure 2. This number will allow for a broad representation across multiple specialties and geographies. There will be a centralised oversight of invitations of panellists meeting the selection criteria.

Supplementary Figure 2: Sampling framework for the Delphi Expert Panel^a,b^

**Footnotes:** ^a^Numbers in brackets, listed after specific countries, show the suggested number of experts per country intended to be recruited. ^b^Experts will be recruited in a manner that maximises geographic diversity in those countries where more than one expert is intended to be recruited.

Additionally, the expertise levels of potential Expert Panellists will be graded according to the criteria outlined in Supplementary Table 1. Each candidate Expert Panellist will be graded as either a Tier 1, Tier 2 or Tier 3 expert, with Tier 1 representing those assigned the highest expertise scores.

Supplementary Table 1: Expertise grading criteria

Leadership

A) Board member on one or more medical association(s)/society(s)

B) Participates regularly as an advisor to industry (e.g. strategy development, advisory boards etc)

C) Current affiliation with top institution in area of specialty as Chief or full Professor at teaching hospital or university

D) Past/current leader of a recognised society/association/agency (e.g. Regulatory body) etc

E) Member in the last 5 years of an executive board or leadership role in an international committee/society or professional medical association

Participation in domestic and/or global speaking engagements during the past 5 years

A) Between 1 to 5 domestic and/or international speaking engagements

B) Between 5 to 10 domestic and/or international speaking engagements

C) More than 10 international speaking engagements

D) No domestic or international speaking engagements

Publications during the past 5 years

A) Published 1–5 peer reviewed articles

B) Published 5–10 peer reviewed articles or participated on 1 journal editorial board or performed systematic reviews

C) (1) Contributed to a textbook (editor or author) AND (2) 10 plus articles for scientific publications in international peer reviewed journals

D) No published articles

Years of relevant postgraduate/residency experience (clinical, industry, other)

A) Less than 5 years

B) Minimum of 5–7 years

C) Minimum of 8–10 years

Recommended or Endorsed by Someone Considered to be a Credible, or Well-Known Thought Leader

Yes/No

Product, treatment or research and development or other relevant experience

A) Experienced

B) Highly Experienced

C) Advanced

D) No relevant experience

Global reputation/recognition amongst peers

Yes/No

Considered a next generation thought leader

Yes/No

2.3.2 Criteria Application Process

Selection of the Expert Panellists will happen as follows:

1. Experts will be assessed against the three key selection criteria listed at the start of this section. Those that meet all criteria will proceed
2. Experts will be assigned to different regions, based on the country that they are licensed and actively practicing within (Asia, the Americas or Europe), and will be ranked based on the tiered grading system
3. Tier 1 experts will be assigned to fill the priority country quotes, per region, outlined in Supplementary Figure 2. Should a priority country lack a Tier 1 expert, Tier 2 experts will be prioritised next, followed by Tier 3. Within each Tier, prioritisation will happen according to score
4. Once all priority countries have been assigned an expert, an assessment of specialism representation will be undertaken. All surgical specialties of interest should be generally represented at a Global level. In the unlikely event that specialty is not represented once all surgeons have been selected, the highest ranking expert for the missing surgical specialty will be recruited as an additional expert, regardless of geographic location

2.3 References

1. Gattrell WT, Logullo P, van Zuuren EJ, Price A, Hughes EL, Blazey P et al. ACCORD (ACcurate COnsensus Reporting Document): A reporting guideline for consensus methods in biomedicine developed via a modified Delphi. *PLoS Med* 2024;21:e1004326
2. Nasa P, Jain R, Juneja D. Delphi methodology in healthcare research: How to decide its appropriateness. *World J Methodol* 2021;11:116-29
3. Ogbeifun E, Agwa-Ejon J, Mbohwa C, Pretorius JHC. The Delphi technique: A credible research methodology. In: Proceedings of the International Conference on Industrial Engineering and Operations Management; Kuala Lumpur, IEOM 2016. IEOM Society; 2016 p.2004-2009
4. Jünger S, Payne SA, Brine J, Radbruch L, Brearley SG. Guidance on Conducting and REporting DElphi Studies (CREDES) in palliative care: Recommendations based on a methodological systematic review. *Palliat Med* 2017;31:684-706

Appendix 3: ACCORD Checklist

Supplementary Table 2: ACCORD checklist

| **Item No.** | **Section** | **Checklist Item (*help text*)** | **Section in Manuscript** |
| --- | --- | --- | --- |
| T1 | Title | Identify the article as reporting a consensus exercise and state the consensus methods used in the title.  *For example, Delphi or nominal group technique.* | N/A: Title omits explicit reference to modified Delphi study for brevity, and because this study is not the sole focus of the paper |
| I1 | Introduction | Explain why a consensus exercise was chosen over other approaches. | Appendix 2; Discussion: Strengths and Limitations |
| I2 |  | State the aim of the consensus exercise, including its intended audience and geographical scope (national, regional, global). | Introduction; Appendix 2 |
| I3 |  | If the consensus exercise is an update of an existing document, state why an update is needed, and provide the citation for the original document. | N/A |
| M1 | Methods  Registration | If the study or study protocol was prospectively registered, state the registration platform and provide a link. If the exercise was not registered, this should be stated.  *Recommended to include the date of registration.* | Appendix 2 |
| M2 | Selection of SC and/or panellists | Describe the role(s) and areas of expertise or experience of those directing the consensus exercise.  *For example, whether the project was led by a chair, co-chairs or a steering committee, and, if so, how they were chosen. List their names if appropriate, and whether there were any subgroups for individual steps in the process.* | Appendix 2 |
| M3 |  | Explain the criteria for panellist inclusion and the rationale for panellist numbers. State who was responsible for panellist selection. | Appendix 2 |
| M4 |  | Describe the recruitment process (how panellists were invited to participate).  *Include communication/advertisement method(s) and locations, numbers of invitations sent, and whether there was centralized oversight of invitations or if panellists were asked/allowed to suggest other members of the panel.* | Appendix 2 |
| M5 |  | Describe the role of any members of the public, patients or carers in the different steps of the study. | N/A |
| M6 | Preparatory research | Describe how information was obtained prior to generating items or other materials used during the consensus exercise.  *This might include a literature review, interviews, surveys, or another process.* | Methods: Evidence-based Statements; Appendix 1 |
| M7 |  | Describe any systematic literature search in detail, including the search strategy and dates of search or the citation if published already.  *Provide the details suggested by the reporting guideline PRISMA and the related PRISMA-Search extension.* | Appendix 1 |
| M8 |  | Describe how any existing scientific evidence was summarized and if this evidence was provided to the panellists. | Methods: Evidence-based Statements; Appendix 1 |
| M9 | Assessing consensus | Describe the methods used and steps taken to gather panellist input and reach consensus (for example, Delphi, RAND-UCLA, nominal group technique).  *If modifications were made to the method in its original form, provide a detailed explanation of how the method was adjusted and why this was necessary for the purpose of your consensus-based study.* | Appendix 2 |
| M10 |  | Describe how each question or statement was presented and the response options. State whether panellists were able to or required to explain their responses, and whether they could propose new items.  *Where possible, present the questionnaire or list of statements as supplementary material.* | Appendix 2; Appendix 5 |
| M11 |  | State the objective of each consensus step.  *A step could be a consensus meeting, a discussion or interview session, or a Delphi round.* | Appendix 6 |
| M12 |  | State the definition of consensus (for example, number, percentage, or categorical rating, such as ‘agree’ or ‘strongly agree’) and explain the rationale for that definition. | Appendix 2 |
| M13 |  | State whether items that met the prespecified definition of consensus were included in any subsequent voting rounds. | Appendix 2; Appendix 6; Results: Final Delphi round and final SSO classification |
| M14 |  | For each step, describe how responses were collected, and whether responses were collected in a group setting or individually. | Appendix 2 |
| M15 |  | Describe how responses were processed and/or synthesized.  *Include qualitative analyses of free-text responses (for example, thematic, content or cluster analysis) and/or quantitative analytical methods, if used.* | Appendix 2 |
| M16 |  | Describe any piloting of the study materials and/or survey instruments.  *Include how many individuals piloted the study materials, the rationale for the selection of those individuals, any changes made as a result and whether their responses were used in the calculation of the final consensus. If no pilot was conducted, this should be stated.* | N/A |
| M17 |  | If applicable, describe how feedback was provided to panellists at the end of each consensus step or meeting.  *State whether feedback was quantitative (for example, approval rates per topic/item) and/or qualitative (for example, comments, or lists of approved items), and whether it was anonymized.* | Appendix 2 |
| M18 |  | State whether anonymity was planned in the study design. Explain where and to whom it was applied and what methods were used to guarantee anonymity. | Appendix 2 |
| M19 |  | State if the steering committee was involved in the decisions made by the consensus panel.  *For example, whether the steering committee or those managing consensus also had voting rights.* | Appendix 2 |
| M20 | Participation | Describe any incentives used to encourage responses or participation in the consensus process.  *For example, were invitations to participate reiterated, or were participants reimbursed for their time.* | Disclosures |
| M21 |  | Describe any adaptations to make the surveys/meetings more accessible.  *For example, the languages in which the surveys/meetings were conducted and whether translations or plain language summaries were available*. | N/A |
| R1 | Results | State when the consensus exercise was conducted. List the date of initiation and the time taken to complete each consensus step, analysis, and any extensions or delays in the analysis. | Supplementary Figure 1 |
| R2 |  | Explain any deviations from the study protocol, and why these were necessary.  *For example, addition of panel members during the exercise, number of consensus steps, stopping criteria; report the step(s) in which this occurred.* | N/A |
| R3 |  | For each step, report quantitative (number of panellists, response rate) and qualitative (relevant socio-demographics) data to describe the participating panellists. | Results: Final Delphi round and final SSO classification; Appendix 6 |
| R4 |  | Report the final outcome of the consensus process as qualitative (for example, aggregated themes from comments) and/or quantitative (for example, summary statistics, score means, medians and/or ranges) data. | Results: Final Delphi round and final SSO classification; Appendix 6 |
| R5 |  | List any items or topics that were modified or removed during the consensus process. Include why and when in the process they were modified or removed. | Appendix 6 |
| D1 | Discussion | Discuss the methodological strengths and limitations of the consensus exercise.  *Include factors that may have impacted the decisions (for example, response rates, representativeness of the panel, potential for feedback during consensus to bias responses, potential impact of any non-anonymized interactions).* | Discussion |
| D2 |  | Discuss whether the recommendations are consistent with any pre-existing literature and, if not, propose reasons why this process may have arrived at alternative conclusions. | Discussion |
| O1 | Other information | List any endorsing organizations involved and their role. | Funding; Disclosures |
| O2 |  | State any potential conflicts of interests, including among those directing the consensus study and panellists. Describe how conflicts of interest were managed. | Disclosures |
| O3 |  | State any funding received and the role of the funder.  *Specify, for example, any funder involvement in the study concept/design, participation in the steering committee, conducting the consensus process, funding of any medical writing support. This could be disclosed in the methods or in the relevant transparency section of the manuscript. Where a funder did not play a role in the process or influence the decisions reached, this should be specified.* | Appendix 2; Funding; Author’s contributions |

Appendix 4: Validation Study Protocol

4.1 Introduction

This protocol describes in detail the proposed analysis approach for this validation study. Specifically outlined within this document are the background and rationale; objectives; a summary of the study design and participant recruitment; the validation methodology, including the case materials, training materials and data collection, as well as the analysis approach.

4.1.1 Background

Surgical site infection (SSI) is one of the most common healthcare-associated infections globally, alongside pneumonia and urinary tract infection.^1^ The definitions, gradings and guidelines advocated by the US Centers for Disease Control and Prevention (CDC) for SSIs are the most commonly used.^2^ However, they rely heavily on subjective clinical judgement and are variable across institutions, which may result in the failure to identify minor yet clinically significant wound presentations causing inconsistencies in reporting.^2,3^ The few others that exist are also applied clinically by varying degrees, such that a universal, pan-specialty definition of SSI remains elusive.^4,5^ Further, there are other surgical site outcomes (SSOs), comprising dehiscence, seroma and haematoma, which the CDC classification system does not recognise,^2^ and existing definitions for these outcomes are equally variable.^6-11^ All SSOs can be formidable barriers to the healing of surgical incisions and can occur independently or as a result of each other.

Previous attempts at scoring systems for SSOs have not had sustained wide acceptance.^12-15^ For a SSO classification to succeed, it needs accurate, universally-applicable definitions across different surgical subspecialities, in order to grade severity and ultimately improve reporting, surveillance, and management of SSOs. It must also be easily understood by all members of the patient team, including non-surgical healthcare professionals, to enable SSOs to be graded consistently by all clinicians, not just surgeons.

4.1.2 DISH Classification System

A comprehensive classification system that defines all of these postoperative surgical outcomes (dehiscence [D], infection [I], seroma [S], and haematoma [H]), has been developed by a five-member Steering Committee and an international panel of experts via a modified Delphi study, and is known as the DISH Classification System. The system encompasses definitions for each outcome (‘D’, ‘I’, ‘S’ and ‘H’), gradings of severity for clinical management, and gradings of severity for clinical presentation.

4.1.3 Validation Study

Following consensus on the composition of the DISH Classification System, the next step is to validate the classification system. The validation is to ensure the DISH Classification System is fit for purpose across all surgical specialties, and can be understood and applied consistently by all relevant members of the surgical care team. Two preliminary demonstration exercises have been undertaken in 2025 in the US and Thailand, whereby healthcare professionals were trained with example case studies before they independently graded a predefined set of surgical outcomes in AI-generated case notes. The learnings from these exercises have informed this protocol. This validation study will be undertaken in February 2026 in Madrid, Spain, as part of a global meeting of experts on surgical outcomes.

4.2 Key Objectives

The primary objective of this validation study is to evaluate the reliability of the DISH Classification System using a predefined set of synthetic surgical case studies. Two validation sessions will take place over two days. Concordance via both inter-rater and intra-rater agreement, and agreement with a reference Steering Committee’s score for each case, will be assessed. Each case note represents a patient with between 0 and 2 surgical site outcome(s) that will be independently graded by surgeons from a wide range of specialties, as well as infection control and prevention specialists, and nurses with expertise in wound care from a wide range of geographies. The face validity of the case notes will be valuated and the clinical plausibility assessed by the Steering Committee ahead of use.

4.3 Methods

4.3.1 Study Design

4.3.1.1 Study Type

This is a global, cross-sectional validation study.

4.3.1.2 Study Location

The validation study will take place in Madrid, Spain, on 19-20 February 2026, as part of a global meeting of experts on surgical outcomes.

4.3.1.3 Study Participants

Participants in the study are attendees at the 2026 Global Surgical Outcomes Symposium. The participants are either surgeons (from across multiple specialties), nurse practitioners or healthcare administrators.

4.3.1.4 Participant Recruitment

**Eligibility Criteria:**

All participants must meet the following criteria:

1. Be able to commit to the time required to participate in the validation study
2. Understand the study contents and provide constructive and critical feedback in English

Work in one of the priority surgical areas (colorectal, hepatobiliary, general, orthopaedic [specialising in total joint arthroplasty], cardiovascular, gynaecology, plastic, spine or thoracic) and/or work in wound care or surgical site infection management.

Considering the global relevance and cross-specialist output of this validation study, a unique sampling framework is required. The objective of the framework proposed in this protocol is to ensure diversity in terms of geography and relevant expertise. The current approach prioritises geographic representation, as any proposed clinical universal classification should be suitable to be used globally, while still ensuring key specialisms are represented at a global level. Prioritisation of geographic diversity was informed by the fact that any proposed clinical universal classification should be designed to be surgical site agnostic, as grading is based on management decisions rather than clinical measures.

To ensure geographic diversity, up to 71 participants will be purposefully recruited from G20 countries across the Americas, Europe, and the Asia-Pacific region.

Members of the Steering Committee are not eligible for participation, due to their involvement in the study design and development process.

**Informed Consent:**

Participants will be required to complete an informed consent form to ensure they are free and autonomous in their decision to participate in the research study.

4.3.1.5 Blinding

Participants will complete the validation sessions independently, therefore will be blinded to other participants’ responses, to prevent being influenced by others. The participants will remain pseudo-anonymised to the study organisers, with only their specialty or focus area, years of experience, region (e.g. EMEA), prior experience with the scale, and whether they are a native English speaker visible in their responses. They will not be asked for their name or institutional affiliation.

4.3.2 Validation Methodology

4.3.2.1 Case Materials

The synthetic patient case notes used in the validation are generated using generative artificial intelligence (Gen-AI); no real patient data are used; each case note was reviewed by clinical domain experts to confirm clinical plausibility and alignment with the vignette specifications before inclusion in the study. These notes are intended to assess the internal validity of the scale, but they may not fully reproduce the cognitive load and ambiguity of real clinical encounters that would be required for external validation, and likely represent a “best case” scenario of case documentation (i.e. cleaner, more complete, and less ambiguous than typical real-world notes).

The case note generation workflow consists of:

1. **Construction of the vignette library and sampling**

Clinical vignettes are a well-established method for evaluating clinician judgment and rating consistency under standardised conditions. Vignettes enable control of case content and ensure coverage of rating categories, which is particularly suitable for inter-rater and intra-rater reliability assessment.

Vignette selection was performed using constrained random sampling without replacement with forbidden-combination filtering from domain-specific strata, followed by coverage validation at the end of each sampling attempt. The algorithm ensures comprehensive coverage while maintaining clinical plausibility.

2. **Construction of the Vignette Universe**

The vignette universe was created by enumerating all allowed combinations across the 8 dimensions (4 domains × 2 categories: Presentation and Intervention).

**Hard Rule 1: Within-Domain Allowed (Presentation × Intervention) Combinations**

For each domain, only clinically plausible (P, I) pairs are permitted.

Supplementary Table 3: Allowed Dehiscence pairs

| D | 0 | 1 | 2 | 3 | 4 |
| --- | --- | --- | --- | --- | --- |
| 0 | + | x | x | x | x |
| A | x | + | + | x | x |
| B | x | x | + | + | x |
| C | x | x | x | + | + |
| D | x | x | x | + | + |

Rows represent Presentation levels (0, A–D) and columns represent Intervention levels (0–4).
A "+" indicates an allowed pair; "×" indicates a disallowed pair.

Supplementary Table 4: Allowed Infection - Inflammation pairs

| I | 0 | 1 | 2 | 3 | 4 |
| --- | --- | --- | --- | --- | --- |
| 0 | + | x | x | x | x |
| A | x | + | + | + | x |
| B | x | x | x | + | + |
| C | x | x | x | x | + |
| D | x | x | x | x | + |

Rows represent Presentation levels (0, A–D) and columns represent Intervention levels (0–4).
A "+" indicates an allowed pair; "×" indicates a disallowed pair.

Supplementary Table 5: Allowed Seroma pairs

| **S** | 0 | 1 | 2 | 3 | 4 |
| --- | --- | --- | --- | --- | --- |
| **0** | + | x | x | x | x |
| A | x | + | + | + | x |
| B | x | + | + | + | x |
| C | x | x | x | + | + |
| D | x | x | x | + | + |

Rows represent Presentation levels (0, A–D) and columns represent Intervention levels (0–4).
A "+" indicates an allowed pair; "×" indicates a disallowed pair.

Supplementary Table 6: Allowed Haematoma pairs

| **H** | 0 | 1 | 2 | 3 | 4 |
| --- | --- | --- | --- | --- | --- |
| 0 | + | x | x | x | x |
| A | x | + | + | x | x |
| B | x | + | + | + | + |
| C | x | x | x | + | + |
| D | x | x | x | x | + |

Rows represent Presentation levels (0, A–D) and columns represent Intervention levels (0–4).
A "+" indicates an allowed pair; "×" indicates a disallowed pair.

**Hard Rule 2: Forbidden Cross-Domain Combinations**

The following cross-domain rules were enforced based on Medical Affairs guidance to prevent clinically implausible severity mismatches and intervention conflicts between co-occurring complications:

**Presentation dimension:**

- Seroma P=1 (minimal) incompatible with Dehiscence P=3,4 (moderate/severe)
- Seroma P=4 (severe) incompatible with Dehiscence P=3,4 (moderate/severe)
- Haematoma P=1 (minimal) incompatible with Dehiscence P=3,4 (moderate/severe)

**Intervention dimension:**

- Infection I=4 incompatible with Dehiscence I=1,2
- Infection I=3 incompatible with Dehiscence I=3,4
- Seroma I=4 incompatible with Dehiscence I=1,2
- Seroma I=3 incompatible with Dehiscence I=3,4
- Haematoma I=4 incompatible with Dehiscence I=1,2
- Haematoma I=3 incompatible with Dehiscence I=3,4

**Universe Size After Filtering:**

Starting from all possible (Hard-Rule 1) two-domain combinations (with up to two active domains per vignette):

- Rows before Hard Rule 2: 466
- Rows after Hard Rule 2: 382
- Removed by Hard Rule 2: 84 (18.03%)

The final admissible universe contains 382 unique vignette configurations.

4.3.2.2 Sampling Algorithm

**Initialisation:**
For each domain (D, I, S, H), all allowed (Presentation, Intervention) pairs are organised into domain-specific strata. These strata are then combined into a single sampling bucket; as each new vignette is constructed, sampling proceeds from the remaining (unselected Presentation and Intervention) pairs in the bucket.

**Iterative vignette construction:**
For each vignette (rows 1 to n), sampling is performed from the remaining pool of (P, I) pairs that have not yet been selected in the current sampling attempt:

1. First domain selection: All remaining (P, I) pairs across all four domain strata are pooled. One pair is randomly selected and its source domain identified
2. First pair removal: The selected (P, I) pair is removed from its domain stratum to prevent reuse within the current attempt
3. Second domain selection: All remaining (P, I) pairs from the three other domains (excluding the first selected domain) are pooled. One pair is randomly selected
4. Second pair removal: The selected (P, I) pair is removed from its domain stratum
5. Vignette assembly: The two selected pairs are assigned to their respective domain columns (e.g., D_P, D_I for Dehiscence; I_P, I_I for Infection). All non-selected domains are set to (0, 0), ensuring each vignette contains up to two active domains (as P=0, I=0 is also a valid combination in the bucket)

**Termination and validation:**
The process continues until *n* vignettes are generated. The algorithm then verifies that:

1. Coverage constraints are satisfied: All allowed within-domain (P, I) pairs appear at least once across the selected set
2. No forbidden cross-domain combinations are present in the final set

If either condition is violated, the entire selection is restarted with the sampling bucket reset.

**Balance Assessment:**
Once a candidate set passes the hard coverage constraints, its representativeness relative to the admissible universe is evaluated. This assessment examines:

- Marginal distributions: For each of the 8 variables (D_P, D_I, I_P, I_I, S_P, S_I, H_P, H_I), the proportion of each level (0–4) in the selected sample is compared to the universe proportions
- Pairwise joint distributions: For all $\binom{8}{2}=28$ variable pairs (D_P vs I_P, D_P vs I_I, etc.) joint frequency tables are constructed for both the universe and the selected sample. The maximum absolute difference in cell proportions is calculated for each pair
- Pairing types: Pairwise comparisons are categorised as:
  - Within-domain (e.g., D_P vs. D_I): Presentation-Intervention relationships within a single complication
  - Cross-domain (e.g., D_P vs. I_P, S_I vs. H_I): Relationships between different complications

The maximum absolute difference across all pairwise cell proportions serves as the primary balance metric. If this metric exceeds a pre-specified threshold (>15%), then:

- We may resample with the same *n* to find a better-balanced candidate, or
- Reformulate the sampling algorithm, increasing the sample size (*n)* to allow greater flexibility in matching universe proportions

The final selected set is chosen as the one with the smallest *n* that satisfies all hard constraints and achieves acceptable balance (maximum absolute difference <15%).

4.3.3 Generation of Simulated Case Notes

First here are the general principles for Gen-AI prompts. They are designed to enforce:

- Strict adherence to the specification
- Consistent terminology and formatting across cases
- Versioning with model version, prompt version, and case ID recorded

Patient case notes are generated using Gen-AI and inputs from medical experts following these steps:

1. Gen-AI produces relevant sentences per domain (intervention and presentation). Total 80 sentences were generated, with 10 per domain - 4 (DISH) x 2(Intervention and Presentation) x 10
2. A Gen-AI self-evaluation pipeline is designed to identify the top three sentences per domain based on predefined criteria. Selected sentences are then evaluated, validated and corrected/edited by medical experts.
3. Gen-AI composes 3 medical notes per patient using the sentences from Step 2, yielding 3*n notes for the n-patient synthetic cohort
4. One validated case note per patient is chosen by the study team. The chosen case notes undergo human Quality Assurance (QA) to confirm internal coherence, clinical plausibility, alignment to intended DISH targets, and absence of identifying content. Where needed, minor edits are made; otherwise, targeted prompt refinements are applied to regenerate and re-assess the note
5. Case notes that pass this QA process are used for validation

An example case note can be found in Appendix 8.

4.3.3.1 Training Materials

Before the validation study, participants will undergo a training exercise, which will be formed of two parts. Firstly, the use of the DISH Classification System will be demonstrated to them by a member of the Steering Committee using five sample cases, which will be worked through as a group. Secondly, the participants will be given an additional five sample cases to work through in small groups, with Steering Committee members available to provide support where needed. The 10 training cases will be produced using the same Gen-AI-based note generation and human QA workflow described above, but drawn from a separate sampling run. These cases will be manually selected to ensure appropriate coverage for training purpose. There will also be an opportunity for participants to discuss and ask questions about the scale before completing the exercises. During the training and main validation session, participants will be provided with printed copies of the DISH Classification scale and the schematic for how to use the scale. Participants will also be provided with printed copies of all cases to be used in the validation study.

4.3.3.2 Data Collection

**Duration of study period:**

The study period will be from the date of the first data collection (estimated Thursday 19^th^ February 2026; first validation session) to the date of the final data collection (estimated Friday 20^th^ February 2026; second validation session) which will be considered as the study completion date.

**Data source:**

Data will be collected via a Microsoft Forms document, which will handled and pseudo-anonymised by a third party independent of the sponsor (Costello Medical), prior to data analysis.

4.3.3.3 Data Format

For each case, participants will rate both the clinical intervention and then clinical presentation (if applicable) for each of the domains. The data will be presented in a tabulated format with a row per participant response. Data will be collected for each case, according to the preset 0-4 (intervention) and a-d (presentation, if intervention≠0) options.

4.3.3.4 Data Variables

In the Microsoft Forms document where data will be collected, various covariates will be captured to allow for sensitivity analyses to be conducted. Participants will be asked to add their role (e.g., surgeon), years of experience, their region (e.g., EMEA) and whether they are a native English speaker but will not be asked for their name or institutional affiliation. They will also be asked whether they have had any prior experience with the scale (e.g., participating in either of the preliminary demonstration exercises at the US or APAC events).

4.3.3.5 Analysis Approach

**Software:**

Analysis will be conducted using Python and R.

**Data Cleaning:**

Not applicable as all collected will be analysed. Other than data formatting, no data manipulation such as deleting records will be performed.

**Handling of Missing Data:**

Not applicable since all case notes will be scored, i.e. there will be no missing responses.

**Sample size considerations:**

We anticipate at least moderate-to-good agreement based on prior pilot experiences. The vignette sample size chosen aims to provide reasonably precise estimates rather than hypothesis testing. However, it is also constrained by practical feasibility considerations.

4.3.4 Analyses

4.3.4.1 Validation Endpoints

Primary endpoint: Cross-domain (multivariate) inter-rater agreement across the full DISH profile (all domains jointly).

Secondary endpoint: Domain-level inter-rater reliability, plus intra-rater reliability and accuracy (as applicable).

4.3.4.2 Analysis method

1. Primary (cross-domain) agreement: Inter-rater and intra-rater reliability will be assessed jointly across all DISH domains by treating each patient’s set of domain scores as a multivariate rating profile (vector). A multivariate reliability framework will be applied and an overall cross-domain agreement metric will be reported (e.g., a Generalisability Theory-based coefficient, patient-level double entry interclass correlation coefficient [DE-ICC], or an equivalent multivariate agreement metric appropriate for the rating scale and study design). The specific multivariate agreement metric will be selected within the pre-specified family of approaches based on whether key prerequisites/assumptions are met and on the feasibility of stable estimation given the achieved sample (number of evaluations, dependent on the number of participants and synthetic cases sampled and generated). Selection criteria will also include evaluating the assumptions of each metric-method
2. Secondary (domain-level) reliability: In addition to the primary cross-domain analysis, reliability will also be estimated separately for each domain using established domain-level metrics (e.g., ICC(2,1) and ICC(2,k) from a two-way random-effects model, and/or Kendall’s W as an ordinal agreement measure), to support interpretability and domain-specific reporting
3. Accuracy (agreement with reference standard): In addition to inter-rater reliability, the accuracy of ratings against a pre-defined reference standard for each case will be assessed. Accuracy will be quantified per domain and overall using exact agreement rates, weighted agreement for ordinal categories (e.g., quadratic-weighted kappa), and error magnitude summaries (e.g., mean absolute error)

4.3.4.3 Planned Analyses

Two types of analysis are planned:

1. Base analysis including all participants
2. Sensitivity analyses for participants who:

- Have not participated in a prior validation exercise before (e.g., US or APAC)
- Have prior experience with the scale
- Are native English speakers
- >= x years of healthcare professional (HCP) experience
- Surgeon vs non-surgeon HCPs

4.3.4.4 Exploratory Analyses

Additional analyses outside of planned analyses, such as analysing by surgical specialties or according to region/country of practice may be conducted in an exploratory fashion according to feasibility and will be indicated as such in any subsequent write-up. Additionally, to assess potential fatigue and order effects, raters will be assigned to two groups that receive the same set of cases in opposite order (forward vs reverse sequence) during the first validation session. The groups will rate the opposing sequence during the second validation session. Early vs late cases (e.g., first vs late tertile) will be compared to detect shifts in score distributions.

4.3.5 Ethics

4.3.5.1 Ethical Approval

This protocol will be submitted to Advarra’s Institutional Review Board for independent ethical approval of the study.

4.3.6 References

1. Sartelli M. Healthcare-associated infections in the surgical setting: How to prevent and treat them. *Advances in Human Biology* 2022;12:127-137
2. Mangram AJ, Horan TC, Pearson ML, Silver LC, Jarvis WR, Hospital Infection Control Practices Advisory Committee A. Guideline for prevention of surgical site infection, 1999. Infect Control Hosp Epidemiol 1999;20:247-280
3. Cho JR, Kim D-W, Shin MJ, Kim ES, Kim HB, Kim MH *et al*. Dual Surveillance of Surgical Site Infections Using CDC and ASEPSIS Criteria: Clinical and Economic Outcomes in Colorectal and Small Bowel Surgery. *World J Surg* 2025;49:3097-3105
4. Daniel VT, Hanley AM, Mahmoud BH. 26786 Delphi method study in defining surgical site infection following Mohs surgery. Journal of the American Academy of Dermatology 2021;85:AB25
5. Sawyer RG, Evans HL, Hedrick TL. Technological advances in clinical definition and surveillance methodology for surgical site infection incorporating surgical site imaging and patient-generated health data. *Surgical infections* 2019;20:541-545
6. Damjanov I. Chapter 3 - Hemodynamic Disorders. In: Damjanov I (ed). *Pathology Secrets*. 3rd ed. Mosby; 2009:38–57
7. De Rooij L, Bosmans JWAM, van Kuijk SMJ, Vissers YLJ, Beets GL, van Bastelaar J. A systematic review of seroma formation following drain-free mastectomy. *Eur J Surg Oncol* 2021;47:757-763
8. Dieter S. Treating a post-surgical seroma or hematoma. Last updated 1 October 2024. <https://sanaramedtech.com/blog/treating-post-surgical-seroma-hematoma/> (accessed 27 May 2026)
9. US Department of Health and Human Services. Common Terminology Criteria for Adverse Events (CTCAE) Version 5.0; November 27, 2017. <https://dctd.cancer.gov/research/ctep-trials/for-sites/adverse-events/ctcae-v5-5x7.pdf> (accessed 22 May 2026)
10. Tollefson MK, Boorjian SA, Leibovich BC. Chapter 20 - Complications of the incision and patient positioning. In: Taneja SS (ed). *Complications of Urologic Surgery*. 4th ed. Philadelphia: W.B. Saunders; 2010:225-236
11. Rosen RD, Manna B. Wound dehiscence. In: StatPearls [Internet]. Treasure Island (FL) StatPearls Publishing. Last updated 1 May 2023. <https://www.ncbi.nlm.nih.gov/sites/books/NBK551712/> (accessed 22 May 2026)
12. Akilov FA, Giyasov SI, Mukhtarov ST, Nasirov R, Alidjanov JF. Applicability of the Clavien-Dindo grading system for assessing the postoperative complications of endoscopic surgery for nephrolithiasis: a critical review. *Turk J Urol* 2013;39:153
13. Bailey IS, Karran SE, Toyn K, Brough P, Ranaboldo C Karran SJ. Community surveillance of complications after hernia surgery. *BMJ* 1992;304:469-471
14. De La Rosette JJ, Opondo D, Daels FP, Giusti G, Serrano A, Kandasami SV *et al*. Categorisation of complications and validation of the Clavien score for percutaneous nephrolithotomy. *Eur Urol* 2012;62:246-255
15. Wilson A, Sturridge M, Treasure T, Grüneberg R. A scoring method (ASEPSIS) for postoperative wound infections for use in clinical trials of antibiotic prophylaxis. Lancet 1986;327:311-312

Appendix 5: Targeted Literature Review Results

5.1 Overview

We searched MEDLINE via PubMed and Ovid SP, Embase via Ovid SP, GuidelineCentral, and Google Scholar, up to July 2024. Terms for the outcomes of the proposed universal classification (“dehiscence”, “infection”, “seroma”, “h[a]ematoma”) were paired interchangeably with terms such as “incisions” and “wounds” and “definition”, “classification”, “rating”, “grading”, “taxonomy”, “barrier” and “perception”.

A lack of formal definitions and standardisation was noted, with studies and classifications biased towards incisional infection alone. There was inconsistency in reporting and knowledge between surgical procedures, institutions and personnel. Previous classifications focused on the clinical presentation of incisions, which can be subjective, rather than on the clinical management of an incisional complication which ultimately drives patient outcomes.

5.2 Dehiscence: Current Definitions and Reporting Guidelines

Nine relevant articles were identified which reported current definitions, symptoms and possible interventions of Dehiscence.^1-9^ Multiple publications described the under-reporting of wound dehiscence, primarily being due to the lack of clarity around the definition.^1,4,5^ Some definitions of dehiscence were reported, although varied across publications, with many primarily reporting in relation to abdominal surgery:^1–3, 9^

- The Common Terminology for Adverse Events (CTCAE) of the US Department of Health and Human Services (HHS) describes Dehiscence as ‘*A finding of separation of the approximated margins of a surgical wound*’^9^
- One study by Morgan-Jones *et al.* 2023 described wound dehiscence as separation of closed wound edges after surgery which may be superficial, partial or complete^4^

The literature identified variable definitions for dehiscence, demonstrating the need for standardised definitions to ensure consistent reporting of surgical outcomes.

**References**

1. Sandy-Hodgetts K. Clinical innovation: the Sandy Grading System for surgical wound dehiscence classification—a new taxonomy. *Wounds International* 2017;8:6-11
2. Gillespie BM, Harbeck EL, Sandy-Hodgetts K, Rattray M, Thalib L, Patel B *et al*. Incidence of wound dehiscence in patients undergoing laparoscopy or laparotomy: a systematic review and meta-analysis. *J Wound Care* 2023;32:S31-S43
3. Morgan-Jones R, Sandy-Hodgetts K, Bruwer F, Chabal L, Clegg H, Couch K *et al*. Harmonised glossary of wound care terms. *J Wound Care* 2023;32(Sup7b):1-16
4. Morgan-Jones R, Downie F, Dowsett C, Milne J, Rochon M, Sandoz H *et al*. Consensus Document. *Prevention, identification and management of surgical wound dehiscence (SWD).* Wounds UK, 2023. <https://wounds-uk.com/wp-content/uploads/2023/10/SN23_CD_SWD_WUK-web.pdf> (accessed 22 May 2026)
5. Sandy-Hodgetts K, Carville K, Leslie GD. Determining risk factors for surgical wound dehiscence: a literature review. *Int Wound J* 2015;12:265-275
6. Rosen RD, Manna B. Wound dehiscence. In: StatPearls [Internet]. Treasure Island (FL) StatPearls Publishing. Last updated 1 May 2023. <https://www.ncbi.nlm.nih.gov/sites/books/NBK551712/> (accessed 22 May 2026)
7. Afzal S, Bashir MM. Determinants of wound dehiscence in abdominal surgery in public sector hospital. *Annals of King Edward Medical University* 2008;14:119
8. Mahey R, Ghetla S, Rajpurohit J, Desai D, Suryawanshi S. A prospective study of risk factors for abdominal wound dehiscence. *Int Surg J* 2017;4:24-28
9. US Department of Health and Human Services. Common Terminology Criteria for Adverse Events (CTCAE) Version 5.0; November 27, 2017. <https://dctd.cancer.gov/research/ctep-trials/for-sites/adverse-events/ctcae-v5-5x7.pdf> (accessed 22 May 2026)

5.3 Infection: Current Definitions and Reporting Guidelines

A total of 20 relevant articles were identified which reported definitions, symptoms and possible interventions of Infection.^1-11,14-21^

Many publications reported that barriers to implementing successful surgical site infection (SSI) surveillance programmes are driven by a lack of standardised definitions and a need for alignment in SSI definitions.^3-7^ One Delphi study aimed to define SSI following Mohs surgery, however, the panellists struggled to reach a consensus on the definition, indicating the need for further refinement of the definition of SSI.^14^ Definitions of SSIs were reported in the literature, however, were inconsistent and devised decades ago:^15^

- The Centers for Disease Control and Prevention (CDC) definition for SSIs, used primarily by the American College of Surgeons National Surgical Quality Improvement Program, is the most widely used criterion in diagnosis of Infection, according to Campwala *et al.* 2019^1,12^
- The CTCAE of the US HHS describes wound infection as ‘*A disorder characterised by an infectious process involving the wound’^11^*

Publications highlighted the need to standardise and align the infection definition due to wide diagnostic variability and the subjective nature of identification. Key criteria for identification included: swelling, heat, redness and host immune response.

**References**

1. Campwala I, Unsell K, Gupta S. A comparative analysis of surgical wound infection methods: predictive values of the CDC, ASEPSIS, and Southampton scoring systems in evaluating breast reconstruction surgical site infections. *Plast Surg* 2019;27:93-99
2. Morgan-Jones R, Sandy-Hodgetts K, Bruwer F, Chabal L, Clegg H, Couch K *et al*. Harmonised glossary of wound care terms. *J Wound Care* 2023;32(Sup7b):1-16
3. Li CN, Bonebrake A, Schroeder S, Lam SK, Raskin JS. Challenges in defining surgical-site infections following hydrocephalus surgeries. *Infect Control Hosp Epidemiol* 2023;44:2065-2067
4. Sliepen J, Onsea J, Zalavras CG, Depypere M, Govaert GA, Morgenstern M *et al*. What is the diagnostic value of the Centers for Disease Control and Prevention criteria for surgical site infection in fracture-related infection? *Injury* 2021;52:2879-2885
5. Nota SP, Braun Y, Ring D, Schwab JH. Incidence of surgical site infection after spine surgery: what is the impact of the definition of infection? *Clin Orthop Relat Res* 2015;473:1612-1619
6. Russo PL, Saguil E, Chakravarthy M, Lee KY, Ling ML, Morikane K *et al*. Improving surgical site infection prevention in Asia-Pacific through appropriate surveillance programs: Challenges and recommendation. *Infect Dis Health* 2021;26:198-207
7. Tanner J, Jones LB, Rochon M, Westwood N, Wloch C, Vaja R *et al*. Barriers and facilitators for surgical site infection surveillance for adult cardiac surgery in a high-income setting: an in-depth exploration. *J Hosp Infect* 2023;141:112-118
8. Rosenthal R, Weber WP, Marti WR, Misteli H, Reck S, Dangel M, Oertli D *et al*. Surveillance of surgical site infections by surgeons: biased underreporting or useful epidemiological data? *J Hosp Infect* 2010;75:178-182
9. Wilson M. Understanding the basics of wound assessment. *Wound Essent* 2012;2:8-12
10. Bolton Primary Care Trust NHS. Wound Care Guidelines March 2008. <https://www.scribd.com/document/332394708/Wound-Care-Giudeline-NHS-Bolton-Hospital> (accessed 26 May 2026)
11. US Department of Health and Human Services. Common Terminology Criteria for Adverse Events (CTCAE) Version 5.0; November 27, 2017. <https://dctd.cancer.gov/research/ctep-trials/for-sites/adverse-events/ctcae-v5-5x7.pdf> (accessed 22 May 2026)
12. Berríos-Torres SI, Umscheid CA, Bratzler DW, Leas B, Stone EC, Kelz RR *et al*. Centers for Disease Control and Prevention Guideline for the Prevention of Surgical Site Infection, 2017. *JAMA Surg* 2017;152:784–791
13. Taylor G, McKenzie M, Kirkland T, Wiens R. Effect of surgeon's diagnosis on surgical wound infection rates. *Am J Infect Control* 1990;18:295-299
14. Daniel VT, Hanley AM, Mahmoud BH. 26786 Delphi method study in defining surgical site infection following Mohs surgery. *J Am Acad Dermatol* 2021;85:AB25
15. Sawyer RG, Evans HL, Hedrick TL. Technological advances in clinical definition and surveillance methodology for surgical site infection incorporating surgical site imaging and patient-generated health data. *Surg Infect* 2019;20:541-545
16. Naureckas Li C, Bonebrake A, Mansavage E, Moravec A, Schroeder SK. Advocating for alignment in pediatric cardiothoracic surgical site infection surveillance definitions. *J Pediatr Infect Dis Soc* 2024;13:379-380
17. Hanly AM, Daniel VT, Mahmoud BH. Results of a National Survey on the Definition of Surgical Site Infections After Mohs Micrographic Surgery. *Dermatol Surg* 2023;49:641-644
18. Macefield RC, Blazeby JM, Reeves BC, King A, Rees J, Pullyblank Avery K *et al*. Remote assessment of surgical site infection (SSI) using patient-taken wound images: development and evaluation of a method for research and routine practice. *J Tissue Viability* 2023;32:94-101
19. Westercamp MD, Dudeck MA, Allen-Bridson KE, Konnor R, Edwards JR, Park BJ, Smith RM *et al.* Performance of simplified surgical site infection (SSI) surveillance case definitions for resource-limited settings: Comparison to SSI cases reported to the National Healthcare Safety Network 2013-2017. *Infect Control Hosp Epidemiol* 2020;41:611-613
20. Bruce J, Russell EM, Mollison J, Krukowski ZH. The quality of measurement of surgical wound infection as the basis for monitoring: a systematic review. *J Hosp Infect* 2001;49:99-108
21. Folch EE, Mahajan AK, Oberg CL, Maldonado F, Toloza E, Krimsky WS, Oh S, Bowling MR, Benzaquen S, Kinsey CM, Mehta AC. Standardized definitions of bleeding after transbronchial lung biopsy: a Delphi consensus statement from the Nashville working group. *Chest* 2020;158:393-400

5.4 Seroma: Current Definitions and Reporting Guideline

Thirteen relevant articles were identified which reported current definitions, symptoms and possible interventions of Seroma.^1-13^ Seroma definitions were predominantly reported for breast procedures but have been found to be highly variable across different studies.^2–8^

- The CTCAE of the US HHS describes Seroma as ‘*A finding of tumor-like collection of serum in the tissues’,* with a grading scale (Grade 1–5) related to the clinical presentation and intervention.^1^
- Morales-Conde *et al.* provided a classification system and definitions for seroma, after laparoscopic ventral hernia repair. The system included five grades (Type 0–Type IV) relating to severity of clinical presentation.^9^

The literature identified variable definitions for seroma; the most common definition described it as a collection of fluid/serum in the tissues under the skin.

**References**

1. US Department of Health and Human Services. Common Terminology Criteria for Adverse Events (CTCAE) Version 5.0; November 27, 2017. <https://dctd.cancer.gov/research/ctep-trials/for-sites/adverse-events/ctcae-v5-5x7.pdf> (accessed 22 May 2026)
2. Tollefson MK, Boorjian SA, Leibovich BC. Chapter 20 - Complications of the incision and patient positioning. In: Taneja SS (ed). *Complications of Urologic Surgery.* 4th ed. Philadelphia: W.B. Saunders; 2010:225-236
3. Dieter S. Treating a post-surgical seroma or hematoma. Last updated 1 October 2024. <https://sanaramedtech.com/blog/treating-post-surgical-seroma-hematoma/> (accessed 27 May 2026)
4. De Rooij L, Bosmans JWAM, van Kuijk SMJ, Vissers YLJ, Beets GL, van Bastelaar J. A systematic review of seroma formation following drain-free mastectomy. *Eur J Surg Oncol* 2021;47:757-763
5. Van Bemmel AJM, van de Velde CJH, Schmitz RF, Liefers GJ. Prevention of seroma formation after axillary dissection in breast cancer: A systematic review. *Eur J Surg Oncol* 2011;37:829-835
6. Srivastava V, Basu S, Shukla VK. Seroma formation after breast cancer surgery: what we have learned in the last two decades. *JBC* 2012;15:373-380
7. Kuroi K, Shimozuma K, Taguchi T, Imai H, Yamashiro H, Ohsumi S *et al*. Pathophysiology of seroma in breast cancer. *Breast Cancer* 2005;12:288-293
8. Agrawal A, Ayantunde AA, Cheung KL. Concepts of seroma formation and prevention in breast cancer surgery. *ANZ J Surg* 2006;76:1088-1095
9. Morales-Conde S. A new classification for seroma after laparoscopic ventral hernia repair. *Hernia* 2012;16:261-267
10. Sedaghat S, Schmitz F, Meschede J, Sedaghat M. Systematic analysis of post-treatment soft-tissue edema and seroma on MRI in 177 sarcoma patients. *Surg Oncol* 2020;35:218-223
11. Boostrom SY, Throckmorton AD, Boughey JC, Holifield AC, Zakaria S, Hoskin TL, et al. Incidence of clinically significant seroma after breast and axillary surgery. *J Am Coll Surg* 2009;208:148-150
12. Pope JE. Chapter 1 - Complications of spinal cord stimulation. In: Ranson MT, Pope JE, Deer TR (eds). *Reducing Risks and Complications of Interventional Pain Procedures*. Philadelphia: W.B. Saunders; 2012:3-10
13. Pogson CJ, Adwani A, Ebbs SR. Seroma following breast cancer surgery. *Eur J Surg Oncol* 2003;29:711-717

5.5 Haematoma: Current Definitions and Reporting Guidelines

A total of 20 relevant articles were identified which reported current definitions, symptoms and possible interventions of Haematoma.^1-20^ Complications and definitions are commonly reported in the literature for subdural, epidural or pocket haematoma, which were of limited relevance to this literature review.^2–6^ Existing haematoma grading scales appeared to be primarily designed for (chronic) subdural haematoma.

- The CTCAE of the US HHS describes Haematoma as *‘a disorder characterized by a localized collection of blood, usually clotted, in an organ, space, or tissue, due to a break in the wall of a blood vessel*’, with a grading scale related to the clinical presentation and intervention.^1^
- Additional definitions for haematoma were reported in the literature, with one publication describing it as ‘*a collection of blood outside of blood vessels’.^17^* This publication indicated that this definition of haematoma is *‘generally’* used in clinical practice, emphasising that it is not a standardised way of reporting the outcome.

**References**

1. US Department of Health and Human Services. Common Terminology Criteria for Adverse Events (CTCAE) Version 5.0; November 27, 2017. <https://dctd.cancer.gov/research/ctep-trials/for-sites/adverse-events/ctcae-v5-5x7.pdf> (accessed 22 May 2026)
2. Shimizu Y, Park C, Tokuda K. Gradation density hematoma is a predictor of chronic subdural hematoma recurrence associated with inflammation of the outer membrane. *Clin Neurol Neurosurg* 2020;194:105839
3. Takeuchi K, Yokoyama T, Wada KI, Kumagai G, Kudo H, Asari T *et al*. A new grading of epidural hematoma or scar formation after posterior cervical spine surgery: evaluation of perioperative related factors, distributions, and clinical outcomes after surgery. *Spine Surg Relat Res* 2019;3:285-294
4. Javadi SA, Naderi F, Javadi AM. The optimal surgical approach for treatment of chronic subdural hematoma: questionnaire assessment of practice in Iran and review of literature. *Acta Med Iran* 2015;53:617-621
5. De Sensi F, Miracapillo G, Cresti A, Severi S, Airaksinen KE. Pocket hematoma: a call for definition. *Pacing Clin Electrophysiol* 2015;38:909-913
6. Song J, Tark A, Larson EL. The relationship between pocket hematoma and risk of wound infection among patients with a cardiovascular implantable electronic device: An integrative review. *Heart & Lung* 2020;49:92-98
7. Stanišić M, Pripp AH. A reliable grading system for prediction of chronic subdural hematoma recurrence requiring reoperation after initial burr-hole surgery. *Neurosurgery* 2017;81:752-760
8. Yogendrakumar V, Moores M, Sikora L, Shamy M, Ramsay T, Fergusson D *et al.* Evaluating hematoma expansion scores in acute spontaneous intracerebral hemorrhage: a systematic scoping review. *Stroke* 2020;51:1305-1308
9. Shen J, Xin W, Li Q, Gao Y, Zhang J. A grading system for the prediction of unilateral chronic subdural hematoma recurrence after initial single burr hole evacuation. *Risk Manag Healthc Policy* 2019;179-188
10. Teo KC, Fong SM, Leung WC, Leung IY, Wong YK, Choi OM *et al*. Location-specific hematoma volume cutoff and clinical outcomes in intracerebral hemorrhage. *Stroke* 2023;54:1548-1557
11. Mignucci-Jiménez G, Matos-Cruz AJ, Koskay G, Hanalioglu S, Gonzalez-Romo NI, Xu Y *et al*. Modified Puerto Rico Recurrence Scale for chronic subdural hematomas: augmenting the grading scale with postoperative pneumocephalus volume. *Acta Neurochirurgica* 2023;165:3229-3238
12. Ragaee MA, Mahmoud RN, Alghriany MA, Abd El-Ghani WM. Isolated traumatic acute subdural haematoma: outcome in relation to age, Glasgow Coma Scale, and haematoma thickness. *Egyptian J of Neurol Psychiatr Neurosurg* 2021;57:1-7
13. Yamamoto T, Katayama Y, Yamamoto T, Kumagawa H, Sugitani M. Features of chronic subdural haematoma developed from definitely identified acute subdural haematoma. *Brain Injury* 1990;4:135-146
14. Mignucci-Jiménez G, Matos-Cruz AJ, Abramov I, Hanalioglu S, Kovacs MS, Preul MC *et al*. Puerto Rico Recurrence Scale: Predicting chronic subdural hematoma recurrence risk after initial surgical drainage. *Surg Neurol Int* 2022;13
15. Schulman S, Kearon C. Definition of major bleeding in clinical investigations of antihemostatic medicinal products in non-surgical patients. *J Thromb Haemost* 2005;3(4):692-694
16. Harding J, Sebag F, Sierra M, Palazzo FF, Henry JF. Thyroid surgery: postoperative hematoma—prevention and treatment. *Langenbecks Arch Surg* 2006;391:169-173
17. Dieter S. Treating a post-surgical seroma or hematoma. Last updated 1 October 2024. <https://sanaramedtech.com/blog/treating-post-surgical-seroma-hematoma/> (accessed 27 May 2026)
18. Tollefson MK, Boorjian SA, Leibovich BC. Chapter 20 - Complications of the incision and patient positioning. In: Taneja SS (ed). *Complications of Urologic Surgery.* 4th ed. Philadelphia: W.B. Saunders; 2010:225-236
19. Megson M. Traumatic subcutaneous haematoma causing skin necrosis. *BMJ Case Rep* 2011:bcr0520114273
20. Damjanov I. Chapter 3 - Hemodynamic Disorders. In: Damjanov I (ed). *Pathology Secrets.* 3rd ed. Mosby; 2009:38–57

5.6 Current Barriers to Reporting Surgical Outcomes and HCP Perceptions of SSIs

Nine relevant articles were identified which reported on healthcare professionals’ (HCPs) perceptions of SSIs and the current barriers to reporting them:^1-9^

- Many studies concluded a need for universal guidelines when reporting SSIs in prospective clinical trials. The reporting of SSIs was found to differ greatly between various institutions and surgical techniques^1-3,8^
- Universal, standardised methods are needed to identify SSIs across all clinical settings^2,3,5^
- One study reported poor knowledge of SSIs among HCPs, highlighting the need for experimental research to evaluate interventions that improve HCPs’ knowledge and attitudes towards SSI surveillance^8^
- Despite there being guidelines in place for reporting and reducing SSIs, they are not being used in clinical practice^1^

There is a clear need for a “common language” for communicating outcomes; language needs to be simple and objective to ensure accuracy and consistency of reporting to enable use and interpretation by all members of the surgical team.

**References**

1. Concepción-González A, Sarmiento JM, Rymond CC, Ezeh C, Sinha R, Lin H *et al*. Evaluating compliance with the 2013 best practice guidelines for preventing surgical site infection in high-risk pediatric spine surgery. *Spine Deform* 2024;12:47-56
2. Dukes KC, Reisinger HS, Schweizer M, Ward MA, Chapin L, Ryken TC *et al*. Examining barriers to implementing a surgical-site infection bundle. *Infect Control Hosp Epidemiol* 2024;45:13-20
3. Tkatschenko D, Hansen S, Koch J, Ames C, Fehlings MG, Berven S *et al*. Prevention of surgical site infections in spine surgery: an international survey of clinical practices among expert spine surgeons. *Global Spine J* 2023;13:2007-2015
4. Maoudah AS, Alshareef L, Babukur RM, Alharthi A, Alnashri BY, Al Shanbari N *et al*. The level of awareness among surgical physicians regarding surgical site infections and the risks associated with wound infections in Makkah. *Cureus* 2023;15:e51111
5. Cherian T, Hedt-Gauthier B, Nkurunziza T, Sonderman K, Gruendl MA, Nihiwacu E, *et al*. Diagnosing post-cesarean surgical site infections in rural Rwanda: development, validation, and field testing of a screening algorithm for use by community health workers. *Surg Infections* 2020;21:613-620
6. Mengesha A, Tewfik N, Argaw Z, Beletew B, Wudu M. Practice of and associated factors regarding prevention of surgical site infection among nurses working in the surgical units of public hospitals in Addis Ababa city, Ethiopia: A cross-sectional study. *PLoS One* 2020;15:e0231270
7. Albishi W, Albeshri MA, Mortada HH, Alzahrani K, Alharbi R, Aljuhani F, Aldaqal S *et al*. Awareness and level of knowledge about surgical site infections and risks of wound infection among medical physicians in King Abdulaziz University Hospital: cross-sectional study. *Interactive J Med Res* 2019;8:e12769
8. Horgan S, Saab MM, Drennan J, Keane D, Hegarty J. Healthcare professionals’ knowledge and attitudes of surgical site infection and surveillance: A narrative systematic review. *Nurse Educ Pract* 2023;69:103637
9. Oliveira MDC, Dalcól C, de Carvalho REFL, Poveda VDB. Patient participation in surgical site infection prevention: perceptions of nurses, physicians and patients. *Rev Esc de Enferm USP* 2023;57:e20220459

| 5.7 Overview of Existing Classification Systems  A wide range of scales and measures to evaluate surgical outcomes were reported in published literature. Twenty-one unique measures for surgical outcomes were found. Limitations were identified for many of the commonly used measures, including their lack of applicability across all surgical specialties. An overview of these measures is shown below. | | |
| --- | --- | --- |
| **Scale** | **Overview of Surgical Outcome Focus** | **Specialty** |
| Clavien-Dindo^1^ | General | All surgeries |
| Centers for Disease Control and Prevention (CDC) Scoring System^2^ | Surgical site infection | All surgeries |
| ASEPSIS^3^ | Wound infection | Primarily abdominal surgery |
| Southampton Wound Score^4^ | Wound infection | Primarily validated in abdominal and colorectal surgery |
| SMArt Wounds Assessment Tool^5^ | Surgical site infection | Orthopedic surgery |
| National Wound Assessment Form (NWAF)^6^ | Surgical site infection | General wound care |
| Applied Wound Management (AWM)^7^ | Surgical site infection | General wound care |
| T.I.M.E. Wound Assessment Tool^8^ | Surgical site infection | General wound care |
| NATVNS Assessment Chart for Wound management^9^ | General (infection, size and depth of wound, exudate amount, tissue type) | General wound care |
| Bates‐Jenson Wound Assessment Tool^10^ | General (infection, size and depth of wound, exudate amount, tissue type) | Chronic wounds and general wound healing |
| Sandy Grading System^11^ | Wound Dehiscence | All surgeries |
| **Scale** | **Overview of Surgical Outcome Focus** | **Specialty** |
| Bolton Hospitals NHS Trust Wound Assessment Chart^12^ | Wound infection | Leg/pressure ulcers and diabetic foot ulcers |
| Sussman Wound Healing Tool (SWHT)^13^ | General wound healing | Pressure ulcer wound healing |
| Sessing Scale^14^ | General wound healing and infection | Pressure ulcer wound healing |
| Pressure Ulcer Scale for Healing (PUSH Tool)^15^ | Size of wound, exudate amount, tissue type | Pressure ulcer wound healing |
| Photographic Wound Assessment Tool (PWAT)^16^ | Size and depth of wound, tissue type | General wounds (requires photograph) |
| Common Terminology for Adverse Events^17^ | Adverse event reporting for dehiscence, infection, seroma and haematoma | All surgeries |
| Definition and Classification of Intraoperative Complications (CLASSIC)^18^ | Intraoperative complications by severity | All surgeries |
| Kaafarani *et al*.^19^ | Intraoperative adverse events | All surgeries |
| Memorial Sloan Kettering Cancer Center’s Surgical Secondary Events Grading System^20^ | Surgical secondary events | Oncology |
| Comprehensive Complication Index (CCI)^21^ | General | Major urological surgery |
| **References**   1. Clavien PA, Barkun J, de Oliveira ML, Vauthey JN, Dindo D, Schulick RD *et al*. The Clavien-Dindo classification of surgical complications: five-year experience. *Ann Surg* 2009;250:187-196 2. Mangram AJ, Horan TC, Pearson ML, Silver LC, Jarvis WR, Hospital Infection Control Practices Advisory Committee A. Guideline for prevention of surgical site infection, 1999. *Infect Control Hosp Epidemiol* 1999;20:247-280 3. Wilson A, Sturridge M, Treasure T, Grüneberg R. A scoring method (ASEPSIS) for postoperative wound infections for use in clinical trials of antibiotic prophylaxis. *Lancet* 1986;327:311-312 4. Bailey IS, Karran SE, Toyn K, Brough P, Ranaboldo C, Karran SJ. Community surveillance of complications after hernia surgery. *BMJ* 1992;304:469-471 5. Kooner SS, Sheehan B, Kendal JK, Johal H. Development of a simple multidisciplinary arthroplasty wound-assessment instrument: the SMArt Wound. *Tool Can J Surg* 2018;61:326 6. Fletcher J. Development of a new wound assessment form. *Wounds UK* 2010;6:92-99 7. Cooper P, Gray D, Russell F, Stringfellow S, Timmons J, Bertram M *et al*. Applied wound management as an audit tool to inform practice. *Wounds UK* 2010;6:66-74 8. Fletcher J. Wound assessment and the TIME framework. *Br J Nurs* 2007;16:462-464 9. NATVNS. Adult wound assessment chart. 2020. <https://www.rightdecisions.scot.nhs.uk/media/22lpnozm/natvns-audlt-wound-assessment-chart-2020694c.pdf> (accessed 26 May 2026) 10. Bates-Jensen B. Bates-Jensen wound assessment tool: Instructions for use. 2001. <https://aci.health.nsw.gov.au/__data/assets/pdf_file/0010/388243/22.-Bates-Jensen-wound-assessment-tool-BWAT.pdf> (accessed 26 May 2026) 11. Sandy-Hodgetts K, Alves P, Conway B, Djohan R, Idensohn P, McIsaac C *et al.* *Optimising prevention of surgical wound complications: detection, diagnosis and prediction.* Wounds International, 2022. <https://woundsinternational.com/consensus-documents/optimising-prevention-surgical-wound-complications-detection-diagnosis-surveillance-and-prediction/> (accessed 26 May 2026) 12. Bolton Primary Care Trust NHS. Wound Care Guidelines March 2008. <https://www.scribd.com/document/332394708/Wound-Care-Giudeline-NHS-Bolton-Hospital> (accessed 26 May 2026) 13. Sussman C, Swanson G. Utility of the Sussman Wound Healing Tool in predicting wound healing outcomes in physical therapy. *Adv Wound Care* 1997;10:74-77 14. Ferrell BA, Artinian BM, Sessing D. The Sessing scale for assessment of pressure ulcer healing. *J Am Geriatr Soc* 1995;43:37-40 15. National Pressure Ulcer Advisory Panel. Pressure Ulcer Scale for Healing (PUSH) Tool 3.0. 1998. <https://learn.npiap.com/products/push-tool-3#tab-product_tab_overview> (accessed 28 May 26) 16. Houghton PE, Kincaid CB, Campbell KE, Woodbury MG, Keast DH. Photographic assessment of the appearance of chronic pressure and leg ulcers. *Ostomy Wound Manage* 2000;46:20-35 17. US Department of Health and Human Services. Common Terminology Criteria for Adverse Events (CTCAE) Version 5.0; November 27, 2017. <https://dctd.cancer.gov/research/ctep-trials/for-sites/adverse-events/ctcae-v5-5x7.pdf> (accessed 22 May 2026) 18. Rosenthal R, Hoffmann H, Clavien PA, Bucher HC, Dell-Kuster S. Definition and classification of intraoperative complications (CLASSIC): Delphi study and pilot evaluation. *World J Surg* 2015;39:1663-1671 19. Kaafarani HM, Mavros MN, Hwabejire J, Fagenholz P, Yeh DD, Demoya M *et al*. Derivation and validation of a novel severity classification for intraoperative adverse events. *J Am Coll Surg* 2014;218:1120-1128 20. Strong VE, Selby LV, Sovel M, Disa JJ, Hoskins W, Dematteo R *et al*. Development and assessment of memorial Sloan Kettering cancer center’s surgical secondary events grading system. *Ann Surg Oncol* 2015;22:1061-1067 21. Staiger RD, Cimino M, Javed A, Biondo S, Fondevila C, Périnel J *et al.* The comprehensive complication index (CCI) is a novel cost assessment tool for surgical procedures. *Ann Surg* 2018;268:784-791 | | |

Appendix 6: Supplementary Results for the Modified Delphi Study Methodology

6.1 Rounds One to Three

A summary of the process, number of statements and consensus outcomes are shown in Supplementary Figure 1. Detailed results for all rounds are provided in Section 5.3.

For Rounds One to Three, there were six clinical presentation and clinical intervention grades (Grade 0–5) that were voted on. The three non-consensus generating scoping statements from Round One were used to gain additional insight and were removed prior to Round Two. Two statements that did not reach consensus in Round One were removed from the process without revision, but their free text responses provided insight for generation of Round Two statements. One statement that did reach consensus in Round One was revised and reissued in Round Two, to reflect the free-text responses and changes made to other statements.

All statements that did not reach consensus in the prior round were revised for the subsequent round. A key area of non-consensus was the definition and grading of infection, which were considered by some to be ambiguous and specialty dependent. In Round One, only 18/32 of the Expert Panel agreed or strongly agreed with the proposed definition for infection. Relevant statements were revised in future rounds to include inflammation as an additional option, and to provide specific markers of infection and defined clinical symptoms. Another common theme of disagreement, which continued into Rounds Two and Three, was the inclusion of antibiotics as a clinical intervention: specifically, which grade they were most appropriate for and whether they should be optional or mandatory. In Round One, 20/32 of respondents agreed or strongly agreed that the clinical intervention of a Grade 2 infection included oral or intravenous antibiotics. However, arguments against this included antibiotics not being essential and, conversely, some felt a more aggressive approach was warranted. Balancing antimicrobial stewardship with the overarching objective of not prescribing a particular intervention was a key area of discussion throughout the Delphi study. Round Three formed the basis of the discussions at the subsequent symposium.

6.2 Post-Round Three Symposium

The first symposium took place in Oxford, UK, 6–8 December 2024, with 37 experts representing 20 countries. Discrepancies in the interpretation of the classification system between the Steering Committee, the Expert Panel and other attendees demonstrated that further refinement of the classification system was required (see Appendix 7).

6.3 Detailed Delphi Panel Results

6.3.1 Delphi Panel Results Round One

Statements in bold are those that did not reach consensus in Round One. Thirty-Two panellists participated in Round One (100% response rate).

Supplementary Table 7: Round One Delphi statements and consensus percentages

| **Statement^a^** | **Consensus, %** |
| --- | --- |
| **Limitations and impact of surgical incision outcome reporting in clinical practice** | |
| 1. The incidence of surgical incision outcomes is likely to be underreported in the community and scientific literature | 97% |
| 2. The establishment of universal guidelines for reporting surgical incision outcomes in prospective clinical trials is essential | 97% |
| 3. Enhanced methods for identifying, quantifying and recording surgical incision outcomes are required | 100% |
| **Current scales to report surgical incision outcomes**  There are existing scales/scores/frameworks/classification systems used in clinical practice which describe surgical incision outcomes, such as the Centers for Disease Control and Prevention (CDC) classification. These existing scales/scores/frameworks/classification systems currently used in clinical practice to report surgical incision outcomes: | |
| 4. Are too generic in describing surgical incision outcomes | 75% |
| 5. **Lack applicability to my surgical specialty, or other specialties and geographies** | **53%** |
| 6. **Consider factors that are not relevant to my practice or clinical setting** | **34%** |
| 7. What specific measures or scales do you currently use in clinical practice for identifying and reporting surgical incision outcomes | Non-consensus generating |
| 8. Considering a universal scale, score or framework for reporting surgical incision outcomes, what are the most and least important aspects to consider? | Non-consensus generating |
| **Definitions** | |
| 9. Dehiscence: The partial or total separation of previously approximated incision edges, or deeper tissues, within a surgical incision site | 94% |
| 10. **Infection: The exhibition of one, or more, of the local signs redness, swelling, heat and/or pain as a likely result of micro-organisms infiltrating the surgical incision site, at any tissue level** | **56%** |
| 11. Seroma: A pocket of clear fluid related to a surgical incision site at any tissue level | 81% |
| 12. Haematoma: Bleeding or accumulation of blood related to the surgical incision site at any tissue level | 81% |
| **Proposed Universal Classification** | |
| 13. These four elements (DISH) broadly encapsulate the key aspects of any surgical incision, irrespective of the site of incision and type of surgery performed | 94% |
| 14. These four elements (DISH) are easily identifiable | 84% |
| 15. These four elements (DISH) can be assessed independently from one another | 81% |
| 16. Beyond DISH, are there any other elements which contribute to outcomes of surgical incision healing that you feel should be reflected in a universal global composite endpoint to measure and report surgical incision outcomes in clinical practice? | Non-consensus generating |
| **Grading Categories: Dehiscence** | |
| 17. The clinical presentation of a Grade 1 dehiscence is "partial separation of the incision edges with no fluid discharge“ | 94% |
| 18. The intervention or clinical management for a Grade 1 dehiscence is "simple surveillance and no need for any intervention” | 75% |
| 19. The clinical presentation of a Grade 2 dehiscence is "partial separation of the incision edges, with minimal fluid discharge“ | 88% |
| 20. The intervention and clinical management for a Grade 2 dehiscence is "cleaning and re-dressing of the incision“ | 81% |
| 21. The clinical presentation of a Grade 3 dehiscence is "substantial separation of the incision edges, without disrupting fascia or the deep tissue“ | 94% |
| 22. The intervention or clinical management for a Grade 3 dehiscence is "re-intervention to re-close the incision using local anesthesia, including usage of advanced incision dressing e.g. VAC therapy in the clinic or operating theatre“ | 75% |
| 23. The clinical presentation of a Grade 4 dehiscence is "extensive separation of the incision edges, involving deep tissue layers“ | 84% |
| 24. The intervention or clinical management for a Grade 4 dehiscence is "surgical re-intervention to irrigate and debride the incision in the operating theatre under general, spinal or regional anesthesia" | 81% |
| 25. The clinical presentation of a Grade 5 dehiscence is "most severe incision dehiscence of all surgical tissue layers with or without organ exposure" | 97% |
| 26. The intervention or clinical management for a Grade 5 dehiscence is "surgical re-intervention in an operating theatre under general, spinal, or regional anesthesia to irrigate, debride and reapproximate the incision (if feasible), including usage of advanced incision dressing e.g. VAC therapy and/or potential reconstructive techniques using tissue flaps and/or skin grafts" | 84% |
| **Grading Categories: Infection** | |
| **27. The clinical presentation of a Grade 1 infection is “one or more of the key signs of infection e.g. redness, swelling, heat or pain are present and related to the incision”** | **66%** |
| **28. The intervention or clinical management for a Grade 1 infection is “simple surveillance and no need for any intervention”** | **66%** |
| **29. The clinical presentation of a Grade 2 infection refers to: “one or more of the key signs of infection e.g. redness, swelling, heat or pain are present and spreading away from the incision but no signs of systemic sepsis”** | **72%** |
| **30. The intervention or clinical management for a Grade 2 infection is "regular surveillance with oral or intravenous antibiotics"** | **63%** |
| 31. The clinical presentation of a Grade 3 infection is "one or more of the key signs of infection e.g. redness, swelling, heat or pain are present, with other localized signs of infection related to the incision e.g. gas in tissues and signs of systemic sepsis" | 81% |
| 32. The intervention or clinical management for a Grade 3 infection is "radiological aspiration, or opening, of the incision, under local anesthetic, to allow drainage of purulent material and gain source control outside of an operating theatre. This may involve use of advanced incision dressings e.g. VAC treatment after drainage. 97%Commencement of a minimum of 72 hours of antibiotics" | 81% |
| 33. The clinical presentation of a Grade 4 infection is "severe infection related to the incision with clear systemic signs of sepsis e.g. fever, raised inflammatory markers" | 97% |
| 34. The intervention or clinical management for a Grade 4 infection is "washout of the incision in an operating theatre. Placement of advanced incision dressing e.g. VAC therapy or VAC and fluid irrigation. Readmission or transfer to critical care setting for management of sepsis" | 88% |
| 35. The clinical presentation of a Grade 5 infection is "septic shock or "sepsis related multi-organ failure" | 94% |
| 36. The intervention or clinical management for a Grade 5 infection is "surgical re-intervention under general, spinal, or regional anesthetic to gain definitive source control e.g. incision debridement, removal of prosthetic material. Medical multi-system approach to treat sepsis with multi-organ failure" | 94% |
| **Grading Categories: Seroma** | |
| 37. The clinical presentation of a Grade 1 seroma is "presence of localized fluid collection related to the surgical incision" | 94% |
| 38. The intervention or clinical management for a Grade 1 seroma is "no need for any intervention or surveillance" | 84% |
| 39. The clinical presentation of a Grade 2 seroma is "presence of localized fluid collection related to the surgical incision with minimal discomfort or pressure in the affected area" | 97% |
| 40. The intervention or clinical management for a Grade 2 seroma is "regular surveillance. Radiological imaging required to confirm seroma" | 78% |
| 41. The clinical presentation of a Grade 3 seroma is "large fluid collection causing moderate discomfort or pressure" | 94% |
| 42. The intervention or clinical management for a Grade 3 seroma is "radiological intervention required to aspirate the seroma. Any intervention is outside of an operating theatre" | 78% |
| 43. The clinical presentation of a Grade 4 seroma is "large, complex fluid collection causing significant symptoms" | 97% |
| 44. The intervention or clinical management for a Grade 4 seroma is "radiological placement of a drain to allow resolution of seroma. Surgical re-intervention to ensure drainage of seroma under local anesthetic in clinic or an operating theatre" | 88% |
| 45. The clinical presentation of a Grade 5 seroma is "large, complex fluid collection causing life-threatening symptoms or signs of becoming a chronic seroma" | 91% |
| 46. The intervention or clinical management for a Grade 5 seroma is "surgical re-intervention, requiring more than local anesthetic, to ensure definitive treatment of seroma in an operating theatre" | 94% |
| **Grading Categories: Haematoma** | |
| **47. The clinical presentation of a Grade 1 haematoma is "purpura related to the incision"** | **68%** |
| 48. The intervention or clinical management for a Grade 1 haematoma is "no need for any intervention or surveillance" | 94% |
| 49. The clinical presentation of a Grade 2 haematoma is "presence of ecchymosis and swelling within the tissues and related to the incision but not causing substantial symptoms to the patient" | 97% |
| 50. The intervention or clinical management for a Grade 2 haematoma is "routine surveillance. Application of pressure dressing" | 94% |
| 51. The clinical presentation of a Grade 3 haematoma is "enlarging haematoma causing swelling and pain" | 97% |
| 52. The intervention or clinical management for a Grade 3 haematoma is "aspiration, or opening, of the incision under local anesthetic to drain haematoma outside of an operating theatre" | 81% |
| 53. The clinical presentation of a Grade 4 haematoma is "significant haematoma causing substantial swelling, pain, and compromising nearby organs or structures" | 94% |
| 54. The intervention or clinical management for a Grade 4 haematoma is "surgical re-intervention, requiring more than local anesthetic, to open incision, remove or wash haematoma and close incision or apply advanced incision dressing e.g. VAC therapy in an operating theatre" | 94% |
| 55. The clinical presentation of a Grade 5 haematoma is "significant haematoma, with active bleeding, causing risk to nearby structures or life" | 97% |
| 56. The intervention or clinical management for a Grade 5 haematoma is "surgical re-intervention, requiring more than local anesthetic, to stop an active bleeding point. Packing of incision for removal at a second re-look of the incision. Application of an advanced incision dressing e.g. VAC therapy. Definitive closure of the surgical incision with pressure dressings" | 88% |

**Footnotes: ^a^**Bold text indicates statement did not reach consensus.

6.3.2 Delphi Results Round Two

Statements in bold are those that did not reach consensus in Round Two. Thirty-one panellists participated in Round Two (97% response rate). One panellist did not vote in Round Two and therefore was removed from voting in subsequent rounds.

Supplementary Table 8: Round Two Delphi statements and consensus percentages

| **Statement^a^** | **Consensus, %** |
| --- | --- |
| 1. INFLAMMATION DEFINITION: Do you agree with the following definition for inflammation? "One or more of the following clinical signs: redness, swelling, heat, pain or serous exudate, related to the incision” | 94% |
| 2. INFECTION DEFINITION: Do you agree with the following definition for infection? "Two or more of the clinical signs (redness, swelling, heat, pain, non-serous exudate), plus one or more of the following signs (elevated C-reactive protein [CRP] levels or an increased white blood cell [WBC] count, or a rise in temperature over 38 degrees Celsius)” | 84% |
| 3. INFLAMMATION/INFECTION: The clinical presentation of a Grade 1 is: “One or more of the following clinical signs of inflammation: redness, swelling, heat, pain or serous exudate, related to the incision” | 90% |
| 4. INFLAMMATION/INFECTION: The intervention or clinical management for a Grade 1 is: “Monitoring and no need for any intervention” | 84% |
| 5. INFECTION: The clinical presentation of a Grade 2 is: “Two or more of the following clinical signs (redness, swelling, heat, pain) plus one or more of the following signs (non-serous exudate, fever, elevated C-reactive protein [CRP] levels or an increased white blood cell [WBC] count, positive wound or blood culture results, or a rise in temperature over 38 degrees Celsius).” | 90% |
| **6. INFECTION: The intervention or clinical management for a Grade 2 is “Regular monitoring with oral or intravenous antibiotics"** | **71%** |
| 7. INFECTION: The clinical presentation of a Grade 3 is: “Two or more of the following clinical signs (redness, swelling, heat, pain) plus one or more of the following signs (non-serous exudate, fever, elevated C-reactive protein [CRP] levels or an increased white blood cell [WBC] count, positive wound or blood culture results, or a rise in temperature over 38 degrees Celsius), with other localized signs of infection related to the incision e.g. gas in tissues and signs of systemic sepsis.” | 77% |
| 8. HAEMATOMA: The clinical presentation of a Grade 1 is “Bruising related to the incision” | 87% |

**Footnotes:** ^a^Bold text indicates statement did not reach consensus.

6.3.3 Delphi Results Round Three

Thirty-One panellists participated in Round Three (100% response rate).

Supplementary Table 9: Round Three Delphi statements and consensus percentages

| **Statement** | **Consensus %** |
| --- | --- |
| 1. INFECTION: The intervention or clinical management for a Grade 2 is: “Regular monitoring with incision management including administration of topical antiseptics and/or systemically administered antibiotics” | 90% |

6.3.4 Delphi Results Round Four

Twenty-nine panellists participated in Round Four (94% response rate). Two participants did not complete Round Four.

Note that Round Four took place after the symposium (see Appendix 7) based on feedback received from participants and their experiences piloting the scale on patient cases.

Supplementary Table 10: Round Four Delphi statements and consensus percentages

| **Statement** | **Consensus %** |
| --- | --- |
| **Objective of DISH** | |
| 1. Please indicate your level of agreement with the following statement describing the objective of the DISH scale:   *‘DISH is an objective classification system, irrespective of clinical setting and surgical specialty, that provides a standardized method to measure, compare and communicate the clinical management of surgical incisions’* | 93% |
| **Structural Changes to the DISH Scale** | |
| 2. Reporting the clinical presentation of an incision independently from the intervention or clinical management allows for comparisons across surgical specialties and settings, while simplifying the DISH scale and grading process. | 97% |
| 3. It would be beneficial to record the "acute" DISH score at different timepoints to demonstrate trends in incision healing or deterioration, as well as the maximum DISH score attained for each individual element (D, I, S and H). | 90% |
| **DEHISCENCE** | |
| 4. CLINICAL INTERVENTION/MANAGEMENT: The intervention or clinical management of “Patient self-monitoring”, indicates Grade 1 | 97% |
| 5. CLINICAL INTERVENTION/MANAGEMENT: The intervention or clinical management of “Local treatment, with or without local anesthesia”, indicates Grade 2 | 97% |
| 6. CLINICAL INTERVENTION/MANAGEMENT: The intervention or clinical management of “Surgical re-intervention with general, spinal or regional anesthesia but without reconstruction”, indicates Grade 3 | 86% |
| 7. CLINICAL INTERVENTION/MANAGEMENT: The intervention or clinical management of “Surgical re-intervention with general, spinal or regional anesthesia and with reconstruction”, indicates Grade 4 | 90% |
| 8. CLINICAL PRESENTATION: The clinical presentation of “Partial separation of the incision edges with no to minimal fluid discharge”, indicates a score of ‘a’ | 97% |
| 9. CLINICAL PRESENTATION: The clinical presentation of “Substantial separation of the incision edges, without disrupting fascia or the deep tissue”, indicates a score of ‘b’ | 93% |
| 10. CLINICAL PRESENTATION: The clinical presentation of “Extensive separation of the incision edges, involving deep tissue layers”, indicates a score of ‘c’ | 97% |
| 11. CLINICAL PRESENTATION: The clinical presentation of “Most severe incision dehiscence of all surgical tissue layers with or without organ exposure”, indicates a score of ‘d’ | 97% |
| **INFLAMMATION/INFECTION** | |
| 12. CLINICAL INTERVENTION/MANAGEMENT: The intervention or clinical management of “Patient self-monitoring”, indicates Grade 1 | 97% |
| 13. CLINICAL INTERVENTION/MANAGEMENT: The intervention or clinical management of “Healthcare professional monitoring; closed incision management; antibiotics optional”, indicates Grade 2 | 83% |
| 14. CLINICAL INTERVENTION/MANAGEMENT: The intervention or clinical management of “Surgical re-intervention with local anesthesia, or percutaneous drainage; antibiotics optional”, indicates Grade 3 | 79% |
| 15. CLINICAL INTERVENTION/MANAGEMENT: The intervention or clinical management of “Surgical re-intervention, with general, spinal or regional anesthesia”, indicates Grade 4 | 86% |
| 16. CLINICAL PRESENTATION: The clinical presentation of “One or more of the following clinical signs of inflammation related to the incision: redness, swelling, heat, pain, or serous exudate”, indicates a score of ‘a’ | 97% |
| 17. CLINICAL PRESENTATION: The clinical presentation of “Two or more of the following clinical signs: redness, swelling, heat, pain, non-serous exudate; plus one or more of the following signs: elevated C-reactive protein or an increased white blood cell count, temperature over 38℃ (100.4℉), positive wound or blood culture, gas in tissues”, indicates a score of ‘b’ | 90% |
| 18. CLINICAL PRESENTATION: The clinical presentation of “Severe infection related to the incision with systemic signs of sepsis or mono-organ failure”, indicates a score of ‘c’ | 86% |
| 19. CLINICAL PRESENTATION: The clinical presentation of “Septic shock; Sepsis related multi-organ failure”, indicates a score of ‘d’ | 79% |
| **SEROMA** | |
| 20. CLINICAL INTERVENTION/MANAGEMENT: The intervention or clinical management of “Patient self-monitoring”, indicates Grade 1 | 93% |
| 21. CLINICAL INTERVENTION/MANAGEMENT: The intervention or clinical management of “Healthcare professional monitoring with or without radiological imaging”, indicates Grade 2 | 93% |
| 22. CLINICAL INTERVENTION/MANAGEMENT: The intervention or clinical management of “Local treatment with local anesthesia, or percutaneous drainage”, indicates Grade 3 | 97% |
| 23. CLINICAL INTERVENTION/MANAGEMENT: The intervention or clinical management of “Surgical re-intervention, with general, spinal or regional anesthesia”, indicates Grade 4 | 97% |
| 24. CLINICAL PRESENTATION: The clinical presentation of “Presence of localized fluid collection related to the surgical incision with no to minimal discomfort or pressure in the affected area”, indicates a score of ‘a’ | 100% |
| 25. CLINICAL PRESENTATION: The clinical presentation of “Large fluid collection causing moderate discomfort or pressure in the affected area”, indicates a score of ‘b’ | 93% |
| 26. CLINICAL PRESENTATION: The clinical presentation of “Large, complex fluid collection causing significant symptoms”, indicates a score of ‘c’ | 86% |
| 27. CLINICAL PRESENTATION: The clinical presentation of “Large, complex fluid collection causing life-threatening symptoms or a chronic walled-off collection”, indicates a score of ‘d’ | 86% |
| **HAEMATOMA** | |
| 28. CLINICAL INTERVENTION/MANAGEMENT: The intervention or clinical management of “Patient self-monitoring”, indicates Grade 1 | 93% |
| 29. CLINICAL INTERVENTION/MANAGEMENT: The intervention or clinical management of “Healthcare professional monitoring”, indicates Grade 2 | 93% |
| 30. CLINICAL INTERVENTION/MANAGEMENT: The intervention or clinical management of “Local treatment with local anesthesia, or percutaneous drainage, or embolization”, indicates Grade 3 | 86% |
| 31. CLINICAL INTERVENTION/MANAGEMENT: The intervention or clinical management of “Surgical re-intervention or radiological intervention with general, spinal or regional anesthesia”, indicates Grade 4 | 97% |
| 32. CLINICAL PRESENTATION: The clinical presentation of “Bruising related to the incision, with or without swelling, but not causing substantial symptoms to the patient”, indicates a score of ‘a’ | 93% |
| 33. CLINICAL PRESENTATION: The clinical presentation of “Enlarging haematoma causing swelling and pain”, indicates a score of ‘b’ | 90% |
| 34. CLINICAL PRESENTATION: The clinical presentation of “Significant haematoma causing substantial swelling, pain, and compromising nearby organs or structures”, indicates a score of ‘c’ | 97% |
| 35. CLINICAL PRESENTATION: The clinical presentation of “Significant haematoma with active bleeding”, indicates a score of ‘d’ | 93% |

Appendix 7: Post-Round Three Symposium

7.1 Critique of the Proposed Classification

The feedback obtained throughout the symposium and during the pilot exercise (see below) provided invaluable insights from the Expert Panel and external attendees and informed subsequent Round Four statements:

- Attendees required clarification of the objective of the classification;
- There was confusion as to whether the score should be made as a ‘one-off’ assessment of an incision or whether several scores could be given to the same incision to observe trends in healing;
- Attendees expressed a preference to visually decouple the clinical presentation and clinical management components of the scale to avoid the inference that the clinical presentation should dictate the clinical management score; and
- There was a preference to simplify the statements within the scale.

In acknowledgement of this feedback, the Steering Committee reassessed the universal classification that reached consensus in Round Three, refining and simplifying the statements prior to Round Four. An objective statement was also devised, to clarify the purpose of the universal classification.

Key limitations in the use of images were noted during the pilot exercise (see below), including the subjective nature of clinical presentation, which can be difficult to discern from a static image. It was also noted that images disproportionately encourage users to focus on clinical presentation, whilst the clinical management of an incision was the driver of patient outcomes. Differences in specialties, approach and technique also meant that the duality between clinical presentation and intervention were interpreted as a prescription of management based on presentation rather than a classification of what had been undertaken.

Subsequently, prior to Round Four voting, the clinical presentation and clinical management components of the scale were separated, to make it clearer that the presentation statements were descriptive only. This aspect of the revision was crucial to ensure the universal classification is applicable to all surgical specialties and represented the most substantial change to the classification system from previous rounds. These changes made it clearer that the DISH score is driven by the clinical management of surgical incisions, whilst the clinical presentation offers useful context.

The newly separated clinical presentation and clinical management scale were both reduced from five to four options per element for clinical presentation and six to five options per element for clinical management. This was in addition to simplifying the statements themselves.

7.2 Pilot exercise of the proposed classification after Round Three (independent of/prior to the formal validation exercise)

The pilot exercise was undertaken at the in-person symposium in Oxford, in December 2024, based on the classification system agreed on following the output of Round Three of the modified Delphi study. At this stage of the process, there were six grades for clinical intervention/management (0–5) and five grades for clinical presentation (a–e). Expert Panel members and non-Expert Panel attendees participated. It is important to note that this was not a validation exercise, instead serving as an opportunity to use the post-Round Three scale to better understand its application and potential limitations when used in practice, and attempt to address any limitations through future work.

The 90-minute exercise comprised 20 anonymised surgical incision photographs, which attendees were required to grade according to the version of the proposed universal classification that had been reached following Round Three of the Delphi study. Attendees used a web-based application to grade the incisions. The results were collated and then compared to the gradings independently agreed by the Steering Committee prior to conduct. A debrief exercise was then undertaken whereby the grades of each decision were discussed in detail.

***Pilot summary and feedback***

- All groups were able to rate all 20 photos
- 2,534 individual data points were collected and informally analysed as a collective
- For 49/80 responses (individual elements), at least 75% of people agreed on the rating given
- Using this threshold for individual elements, however:
  - Dehiscence: 10/20 responses reached consensus (4/14 non-D0 incisions)
  - Infection: 5/20 reached consensus (1/16 non-I0 incisions)
  - Seroma: 15/20 reached consensus (1/4 non-S0 incisions)
  - Haematoma: 19/20 reached consensus (1/2 non-H0 incisions)
- There was a clear learning curve in rating incisions
  - It was noted that a formal validation exercise would make use of a “training set” prior to conduct
  - Time pressure for earlier photos may also have caused some artefacts in rating
- Whilst there was overall low “perfect” consensus, most incisions straddled two grades
  - Individual delegate feedback: “0-5 is easy; 2-4 is difficult”
- A lack of detail in supplied narrative/case notes made scoring challenging in some cases
  - Some feedback noted that scoring would be simpler for their own cases due to familiarity
- There was ambiguity over whether score reflects the current image of the incision or if previous interventions have been described accurately
- There was ambiguity over how prophylactic versus “active” interventions e.g. vacuum-assisted closure and negative pressure wound therapy should be interpreted
- Difficulties were noted in interpreting dehiscence and haematoma independently due to similar interventions

Appendix 8: Validation Case Worked Example

8.1 Example Case Note

Ms. CV is a 66-year-old female (BMI 30.2) with obesity and sleep apnoea who underwent gastrectomy. Early postoperative inspection demonstrated a well-approximated incision with undisturbed edge integrity; no clinically significant dehiscence was present. There was no evidence of postoperative haematoma. She later presented with marked erythema, swelling, and purulent drainage from the incision, accompanied by fever and elevated inflammatory markers, indicating postoperative wound infection. The infection was managed with percutaneous drainage performed under local anaesthesia and initiation of targeted antibiotics based on culture results. In the subsequent postoperative period, a large, complex seroma was identified, causing marked discomfort and swelling. This seroma necessitated surgical exploration and evacuation under general anaesthesia, followed by reconstruction of the wound bed. Post-intervention assessment confirmed that the incision remained well approximated without clinically significant dehiscence, and no postoperative haematoma was observed. Management events were distinct: the infection episode required local anaesthesia-based percutaneous drainage with culture-directed antibiotics, whereas the complex seroma required operative exploration, evacuation, and reconstruction under general anaesthesia. No further intervention was needed for dehiscence or haematoma.

8.2 DISH Scoring Process

The DISH Scoring Process from Figure 1 has been followed and is described in a step-by-step process below.

8.2.1 Dehiscence

**Step 1: Is dehiscence present?**

No, the case note states “a well-approximated incision with undisturbed edge integrity; no clinically significant dehiscence was present.”

**Step 2: What clinical management was employed?**

Not applicable.

**Step 3: What was the clinical presentation?**

Not applicable.

**Step 4: Collate clinical presentation and management score for dehiscence**

D0

8.2.2 Inflammation/Infection

**Step 1: Is inflammation or infection present?**

Yes, the case note reports signs of infection including marked erythema, swelling, purulent drainage accompanied by a fever and elevated inflammatory markers.

**Step 2: What clinical management was employed?**

The infection “was managed with percutaneous drainage performed under local anaesthesia and initiation of targeted antibiotics.” This corresponds to grade 3 of the Clinical Management Component of the DISH Scale (Table 2): “surgical re-intervention with local anaesthesia, or percutaneous drainage; antibiotics optional.”

**Step 3: What was the clinical presentation?**

The clinical presentation of the infection was “marked erythema, swelling, and purulent drainage from the incision, accompanied by fever with elevated inflammatory markers.” This corresponds to grade b from the Clinical Presentation Component of the DISH Scale (Table 3): “two or more of the following clinical signs: redness, swelling, heat, pain, non-serous exudate; plus one or more of the following: elevated C-reactive protein, increased serum white blood cell count, temperature over 38℃ (100.4℉), positive wound or blood culture, gas in tissues.”

**Step 4: Collate clinical presentation and management score for inflammation/infection**

I3b

8.2.3 Seroma

**Step 1: Is seroma present?**

Yes, the case note states “a large, complex seroma was identified.”

**Step 2: What clinical management was employed?**

The seroma “necessitated surgical exploration and evacuation under general anaesthesia, followed by reconstruction of the wound bed.” This corresponds to grade 4 of the Clinical Management Component of the DISH Scale (Table 2): “surgical re-intervention, with general or regional anaesthesia.”

**Step 3: What was the clinical presentation?**

The case note reports that “a large, complex seroma was identified, causing marked discomfort and swelling.” This corresponds to grade c of the Clinical Presentation Component of the DISH Scale (Table 3): “large fluid collection causing significant clinical signs or symptoms.”

**Step 4: Collate clinical presentation and management score for seroma**

S4c

8.2.4 Haematoma

**Step 1: Is haematoma present?**

No, the case note reports “no evidence of postoperative haematoma.”

**Step 2: What clinical management was employed?**

Not applicable.

**Step 3: What was the clinical presentation?**

Not applicable.

**Step 4: Collate clinical presentation and management for haematoma**

H0

8.2.5 Overall DISH Score

After combining the scores for each individual domain, the final overall DISH Score is: **D0 13b S4c H0.**

Appendix 9: Supplementary Validation Results of SSO rating by DISH

**Supplementary Table 11: Validation results heatmaps**

**Management	Presentation**

| #10 |  |  |  |  |  | #10 |  |  |  |  |
| --- | --- | --- | --- | --- | --- | --- | --- | --- | --- | --- |
| 0 | 1 | 118 | 123 | 6 |  |  | 0 | 0 | 0 | 0 |
| 1 | 8 | 3 | 0 | 109 |  | a | 4 | 5 | 0 | 117 |
| 2 | 114 | 2 | 0 | 8 |  | b | 114 | 0 | 0 | 0 |
| 3 | 0 | 0 | 0 | 0 |  | c | 3 | 0 | 0 | 0 |
| 4 | 0 | 0 | 0 | 0 |  | d | 0 | 0 | 0 | 0 |
|  |  |  |  |  |  |  |  |  |  |  |
| #11 |  |  |  |  |  | #11 |  |  |  |  |
| 0 | 0 | 120 | 123 | 4 |  |  | 0 | 0 | 0 | 0 |
| 1 | 0 | 0 | 0 | 1 |  | a | 0 | 1 | 0 | 0 |
| 2 | 0 | 0 | 0 | 1 |  | b | 2 | 0 | 0 | 7 |
| 3 | 51 | 0 | 0 | 0 |  | c | 2 | 2 | 0 | 53 |
| 4 | 72 | 3 | 0 | 117 |  | d | 118 | 0 | 0 | 59 |
|  |  |  |  |  |  |  |  |  |  |  |
| #12 |  |  |  |  |  | #12 |  |  |  |  |
| 0 | 4 | 7 | 122 | 122 |  |  | 0 | 0 | 0 | 0 |
| 1 | 8 | 44 | 1 | 1 |  | a | 118 | 115 | 1 | 1 |
| 2 | 111 | 72 | 0 | 0 |  | b | 1 | 1 | 0 | 0 |
| 3 | 0 | 0 | 0 | 0 |  | c | 0 | 0 | 0 | 0 |
| 4 | 0 | 0 | 0 | 0 |  | d | 0 | 0 | 0 | 0 |
|  |  |  |  |  |  |  |  |  |  |  |
| #13 |  |  |  |  |  | #13 |  |  |  |  |
| 0 | 0 | 123 | 123 | 122 |  |  | 0 | 0 | 0 | 0 |
| 1 | 0 | 0 | 0 | 0 |  | a | 0 | 0 | 0 | 0 |
| 2 | 0 | 0 | 0 | 0 |  | b | 0 | 0 | 0 | 0 |
| 3 | 1 | 0 | 0 | 0 |  | c | 114 | 0 | 0 | 1 |
| 4 | 122 | 0 | 0 | 1 |  | d | 9 | 0 | 0 | 0 |
|  |  |  |  |  |  |  |  |  |  |  |
| #14 |  |  |  |  |  | #14 |  |  |  |  |
| 0 | 123 | 123 | 0 | 1 |  |  | 0 | 0 | 0 | 0 |
| 1 | 0 | 0 | 0 | 0 |  | a | 0 | 0 | 3 | 0 |
| 2 | 0 | 0 | 1 | 1 |  | b | 0 | 0 | 19 | 11 |
| 3 | 0 | 0 | 122 | 1 |  | c | 0 | 0 | 97 | 2 |
| 4 | 0 | 0 | 0 | 120 |  | d | 0 | 0 | 4 | 109 |
| #15 |  |  |  |  |  | #15 |  |  |  |  |
| 0 | 123 | 122 | 0 | 0 |  |  | 0 | 0 | 0 | 0 |
| 1 | 0 | 0 | 0 | 11 |  | A | 0 | 1 | 121 | 3 |
| 2 | 0 | 1 | 4 | 112 |  | B | 0 | 0 | 2 | 115 |
| 3 | 0 | 0 | 119 | 0 |  | C | 0 | 0 | 0 | 5 |
| 4 | 0 | 0 | 0 | 0 |  | D | 0 | 0 | 0 | 0 |
| #16 |  |  |  |  |  | #16 |  |  |  |  |
| 0 | 123 | 123 | 3 | 0 |  |  | 0 | 0 | 0 | 0 |
| 1 | 0 | 0 | 99 | 0 |  | a | 0 | 0 | 119 | 0 |
| 2 | 0 | 0 | 21 | 1 |  | b | 0 | 0 | 0 | 119 |
| 3 | 0 | 0 | 0 | 122 |  | c | 0 | 0 | 0 | 4 |
| 4 | 0 | 0 | 0 | 0 |  | d | 0 | 0 | 0 | 0 |
| #17 |  |  |  |  |  | #17 |  |  |  |  |
| 0 | 123 | 123 | 123 | 123 |  |  | 0 | 0 | 0 | 0 |
| 1 | 0 | 0 | 0 | 0 |  | a | 0 | 0 | 0 | 0 |
| 2 | 0 | 0 | 0 | 0 |  | b | 0 | 0 | 0 | 0 |
| 3 | 0 | 0 | 0 | 0 |  | c | 0 | 0 | 0 | 0 |
| 4 | 0 | 0 | 0 | 0 |  | d | 0 | 0 | 0 | 0 |
| #18 |  |  |  |  |  | #18 |  |  |  |  |
| 0 | 120 | 0 | 5 | 119 |  |  | 0 | 0 | 0 | 0 |
| 1 | 0 | 0 | 3 | 1 |  | a | 0 | 1 | 117 | 2 |
| 2 | 0 | 3 | 111 | 1 |  | b | 0 | 0 | 1 | 0 |
| 3 | 1 | 5 | 2 | 0 |  | c | 2 | 2 | 0 | 0 |
| 4 | 2 | 115 | 2 | 1 |  | d | 1 | 120 | 0 | 1 |

**Management	Presentation**

| #1 | D | I | S | H |  | #1 | D | I | S | H |
| --- | --- | --- | --- | --- | --- | --- | --- | --- | --- | --- |
| 0 | 123 | 12 | 2 | 123 |  |  | 0 | 0 | 0 | 0 |
| 1 | 0 | 107 | 2 | 0 |  | a | 0 | 111 | 3 | 0 |
| 2 | 0 | 3 | 1 | 0 |  | b | 0 | 0 | 27 | 0 |
| 3 | 0 | 1 | 118 | 0 |  | c | 0 | 0 | 91 | 0 |
| 4 | 0 | 0 | 0 | 0 |  | d | 0 | 0 | 0 | 0 |
|  |  |  |  |  |  |  |  |  |  |  |
| #2 |  |  |  |  |  | #2 |  |  |  |  |
| 0 | 122 | 123 | 2 | 1 |  |  | 0 | 0 | 0 | 0 |
| 1 | 1 | 0 | 116 | 105 |  | a | 0 | 0 | 56 | 6 |
| 2 | 0 | 0 | 5 | 16 |  | b | 1 | 0 | 64 | 114 |
| 3 | 0 | 0 | 0 | 1 |  | c | 0 | 0 | 1 | 2 |
| 4 | 0 | 0 | 0 | 0 |  | d | 0 | 0 | 0 | 0 |
|  |  |  |  |  |  |  |  |  |  |  |
| #3 |  |  |  |  |  | #3 |  |  |  |  |
| 0 | 3 | 122 | 1 | 123 |  |  | 0 | 0 | 0 | 0 |
| 1 | 8 | 0 | 1 | 0 |  | a | 6 | 0 | 0 | 0 |
| 2 | 13 | 0 | 0 | 0 |  | b | 111 | 1 | 11 | 0 |
| 3 | 68 | 0 | 0 | 0 |  | c | 3 | 0 | 110 | 0 |
| 4 | 31 | 1 | 121 | 0 |  | d | 0 | 0 | 1 | 0 |
|  |  |  |  |  |  |  |  |  |  |  |
| #4 |  |  |  |  |  | #4 |  |  |  |  |
| 0 | 123 | 122 | 123 | 0 |  |  | 0 | 0 | 0 | 0 |
| 1 | 0 | 1 | 0 | 0 |  | a | 0 | 1 | 0 | 1 |
| 2 | 0 | 0 | 0 | 0 |  | b | 0 | 0 | 0 | 114 |
| 3 | 0 | 0 | 0 | 0 |  | c | 0 | 0 | 0 | 4 |
| 4 | 0 | 0 | 0 | 123 |  | d | 0 | 0 | 0 | 4 |
|  |  |  |  |  |  |  |  |  |  |  |
| #5 |  |  |  |  |  | #5 |  |  |  |  |
| 0 | 116 | 0 | 6 | 123 |  |  | 0 | 0 | 0 | 0 |
| 1 | 0 | 0 | 0 | 0 |  | a | 2 | 0 | 1 | 0 |
| 2 | 0 | 2 | 1 | 0 |  | b | 1 | 102 | 7 | 0 |
| 3 | 1 | 2 | 1 | 0 |  | c | 4 | 18 | 16 | 0 |
| 4 | 6 | 119 | 115 | 0 |  | d | 0 | 3 | 92 | 0 |
|  |  |  |  |  |  |  |  |  |  |  |
| #6 |  |  |  |  |  | #6 |  |  |  |  |
| 0 | 123 | 0 | 123 | 2 |  |  | 0 | 0 | 0 | 0 |
| 1 | 0 | 0 | 0 | 4 |  | a | 0 | 104 | 0 | 120 |
| 2 | 0 | 5 | 0 | 117 |  | b | 0 | 19 | 0 | 1 |
| 3 | 0 | 118 | 0 | 0 |  | c | 0 | 0 | 0 | 0 |
| 4 | 0 | 0 | 0 | 0 |  | d | 0 | 0 | 0 | 0 |
|  |  |  |  |  |  |  |  |  |  |  |
| #7 |  |  |  |  |  | #7 |  |  |  |  |
| 0 | 0 | 122 | 1 | 123 |  |  | 0 | 0 | 0 | 0 |
| 1 | 0 | 0 | 5 | 0 |  | a | 0 | 0 | 15 | 0 |
| 2 | 0 | 1 | 117 | 0 |  | b | 0 | 1 | 105 | 0 |
| 3 | 0 | 0 | 0 | 0 |  | c | 13 | 0 | 2 | 0 |
| 4 | 123 | 0 | 0 | 0 |  | d | 110 | 0 | 0 | 0 |
| #8 |  |  |  |  |  | #8 |  |  |  |  |
| 0 | 123 | 123 | 8 | 1 |  |  | 0 | 0 | 0 | 0 |
| 1 | 0 | 0 | 3 | 0 |  | a | 0 | 0 | 22 | 1 |
| 2 | 0 | 0 | 0 | 1 |  | b | 0 | 0 | 92 | 12 |
| 3 | 0 | 0 | 112 | 121 |  | c | 0 | 0 | 1 | 109 |
| 4 | 0 | 0 | 0 | 0 |  | d | 0 | 0 | 0 | 0 |
|  |  |  |  |  |  |  |  |  |  |  |
| #9 |  |  |  |  |  | #9 |  |  |  |  |
| 0 | 3 | 0 | 123 | 122 |  |  | 0 | 0 | 0 | 0 |
| 1 | 90 | 2 | 0 | 0 |  | a | 109 | 0 | 0 | 0 |
| 2 | 25 | 3 | 0 | 1 |  | b | 11 | 122 | 0 | 0 |
| 3 | 5 | 118 | 0 | 0 |  | c | 0 | 1 | 0 | 1 |
| 4 | 0 | 0 | 0 | 0 |  | d | 0 | 0 | 0 | 0 |

**Management	Presentation**

| #19 | D | I | S | H |  | #19 | D | I | S | H |
| --- | --- | --- | --- | --- | --- | --- | --- | --- | --- | --- |
| 0 | 0 | 0 | 123 | 123 |  |  | 0 | 0 | 0 | 0 |
| 1 | 0 | 2 | 0 | 0 |  | a | 0 | 1 | 0 | 0 |
| 2 | 0 | 1 | 0 | 0 |  | b | 2 | 84 | 0 | 0 |
| 3 | 88 | 5 | 0 | 0 |  | c | 115 | 37 | 0 | 0 |
| 4 | 35 | 115 | 0 | 0 |  | d | 6 | 1 | 0 | 0 |

**Supplementary Table 12:** **Cross-domain per-case DE-ICC**

| Patient | mean DE_ICC (NA excluded) INTER-RATER | mean DE_ICC (NA included) | mean DE_ICC (NA excluded) INTRA-RATER | mean DE_ICC (NA included) |
| --- | --- | --- | --- | --- |
| 1 | 0.9501950 | 0.9501950 | 0.91067 | 0.91067 |
| 2 | 0.8828077 | 0.8828077 | 0.9185883 | 0.9185883 |
| 3 | 0.9306413 | 0.9306413 | 0.9297567 | 0.9297567 |
| 4 | 0.9877645 | 0.9877645 | 0.991445 | 0.991445 |
| 5 | 0.7956711 | 0.7956711 | 0.883675 | 0.883675 |
| 6 | 0.9613938 | 0.9613938 | 0.96865 | 0.96865 |
| 7 | 0.9884269 | 0.9884269 | 0.9883067 | 0.9883067 |
| 8 | 0.9252612 | 0.9252612 | 0.92464 | 0.92464 |
| 9 | 0.9358172 | 0.9358172 | 0.94376 | 0.94376 |
| 10 | 0.9227844 | 0.9227844 | 0.925935 | 0.925935 |
| 11 | 0.9050338 | 0.9050338 | 0.9292283 | 0.9292283 |
| 12 | 0.8338525 | 0.8338525 | 0.88464833 | 0.88464833 |
| 13 | 0.9954977 | 0.9954977 | 0.9872033 | 0.9872033 |
| 14 | 0.9482370 | 0.9482370 | 0.9681333 | 0.9681333 |
| 15 | 0.9720840 | 0.9720840 | 0.9765633 | 0.9765633 |
| 16 | 0.9711531 | 0.9711531 | 0.9787817 | 0.9787817 |
| 17 | NA | 1.0000000 | NA | 1 |
| 18 | 1.0000000 | 1.0000000 | 0.9486767 | 0.9486767 |
| 19 | 0.9762000 | 0.9762000 | 0.97019 | 0.97019 |
| mean | 0.94 | 0.939 | 0.94 | 0.948 |

**Supplementary Table 13:** **Per domain results**

| **Domain** | **ICC2_1_Agreement** | **Kendall's W Coefficient** | **Mean Accuracy** |
| --- | --- | --- | --- |
| **D(I)** | 0.94 | 0.96 | 90% |
| **D(P)** | 0.96 | 0.97 | 96% |
| **H(I)** | 0.96 | 0.96 | 97% |
| **H(P)** | 0.92 | 0.95 | 93% |
| **I(I)** | 0.97 | 0.97 | 96% |
| **I(P)** | 0.96 | 0.97 | 91% |
| **S(I)** | 0.94 | 0.94 | 96% |
| **S(P)** | 0.90 | 0.93 | 87% |
| **Mean** | 0.94 | 0.96 | 93% |

**ICC2_1 Agreement (shows if evaluators gave the same answers, irrespective of the truth):**

- <0.50 = Poor
- 0.50 – 0.75 = Moderate
- 0.75 – 0.90 = Good
- >0.90 = Excellent

**Kendall’s W Coefficient (shows if evaluators ranked the cases similarly [ordinal agreement] across multiple raters):**

- <0.20 = slight
- 0.20 – 0.40 = fair
- 0.40 – 0.60 = moderate
- 0.60 – 0.80 = substantial
- >0.80 = almost perfect

**Mean Accuracy (shows the % correct answers vs the benchmark)**

**Supplementary Table 14:** **Per domain results sensitivity analysis, without zeroes**

| **Domain** | **ICC2_1_Agreement** | **Kendal's W Coefficient** | **Mean Accuracy** |
| --- | --- | --- | --- |
| **D(I)** | 0.84 | 0.86 | 78% |
| **D(P)** | 0.92 | 0.94 | 92% |
| **H(I)** | 0.88 | 0.91 | 94% |
| **H(P)** | 0.78 | 0.83 | 87% |
| **I(I)** | 0.90 | 0.92 | 90% |
| **I(P)** | 0.90 | 0.92 | 77% |
| **S(I)** | 0.77 | 0.84 | 93% |
| **S(P)** | 0.71 | 0.77 | 75% |
| **Mean** | 0.84 | 0.87 | 86% |

**ICC2_1 Agreement (shows if evaluators gave the same answers, irrespective of the truth):**

- <0.50 = Poor
- 0.50 – 0.75 = Moderate
- 0.75 – 0.90 = Good
- >0.90 = Excellent

**Kendall’s W Coefficient (shows if evaluators ranked the cases similarly [ordinal agreement] across multiple raters):**

- <0.20 = slight
- 0.20 – 0.40 = fair
- 0.40 – 0.60 = moderate
- 0.60 – 0.80 = substantial
- >0.80 = almost perfect

**Mean Accuracy (shows the % correct answers vs the benchmark)**

**Supplementary Table 15: Metrics**

| **Metric** | **Definition in English** | **Pros** | **Cons** | **How this maps to primary objective** |
| --- | --- | --- | --- | --- |
| **Accuracy (vs key)** | How often raters match the answer key, optionally giving partial credit when they are close rather than exact | Directly measures validity vs. ground truth | Choice of weights affects magnitude | Compute a single cross-domain accuracy per rater-case (mean across 8 domains) |
| **ICC (2,1)** | ICC (2,1) - 2 way random effect, represent single rater, measures agreement (not just ranking of answers) | Widely recognised; CIs available. | Zero scores problem | Cross domain via average or multivariate or other |
| **Fleiss' kappa** | How much better is the agreement than chance? Weighted versions give more credit to near-misses than far-misses | Chance-corrected agreement; no problem with zero scores | No natural cross-domain | Not multivariate |
| **Gwet’s AC1/AC2** | A chance-corrected agreement measure like kappa, but designed to be more stable when some categories are rare or raters use categories unevenly. AC2 respects ordered distances | A chance-corrected agreement measure; no problem with zero scores | Less familiar to some audiences than kappa/ICC; | Not multivariate |
| **DE-ICC** | A single number that says how similar entire 8‑domain profiles are between raters—rewarding identical overall level and pattern, and penalising systematic “high/low” drift | Captures whole‑profile agreement (level + pattern) in one index | Does not explain if the difference is due to pattern or ranks | Directly targets your goal: agreement on the full 8‑domain vector. More stringent than averaging 8 per‑domain ICCs |
| **G-Theory** | Decomposes observed score variance into components for cases (c), raters (r), domains (d), and their interactions | Rich variance‑component insight (where disagreement comes from) | More complex to explain/run; needs adequate sample size | Exactly made for our objective |

**Supplementary Table 16: DE_ICC Inter-Rater Results**

| **n_patients** | **n_pairs_total** | **Mean_de_icc** | **Median_de_icc** | **Lower_95** | **Upper_95** |
| --- | --- | --- | --- | --- | --- |
| 19 | 35929 | 0.933548 | 1 | 0.9075118 | 0.957657 |

- <0.50 = Poor
- 0.50 – 0.75 = Moderate
- 0.75 – 0.90 = Good
- >0.90 = Excellent

**Supplementary Table 17: DE_ICC Intra-Rater Results**

| **n_patients** | **n_pairs_total** | **Mean_de_icc** | **Median_de_icc** | **Lower_95** | **Upper_95** |
| --- | --- | --- | --- | --- | --- |
| 19 | 1216 | 0.9460473 | 1 | 0.9275589 | 0.962932 |

**Post-Validation Modifications to the DISH Classification System**

- All domain elements mentioning 'general, spinal or regional anaesthesia' were simplified to 'general or regional anaesthesia', as spinal is a form of regional anaesthesia and regional also comprises epidural, for sake of simplification

*Dehiscence (intervention):*

Re-approximation was clarified and a mechanism to classify open wounds was added:

- D3: Surgical re-intervention with general or regional anaesthesia, with simple incisional re-approximation (closure)
- D4: Surgical re-intervention with general or regional anaesthesia, with complex re-approximation (reconstruction) or leaving wound open (no re-approximation or reconstruction)

*Seroma (presentation):*

The word “complex” was removed from grades c and d:

- S c - Large fluid collection causing significant clinical signs or symptoms
  [instead of 'Large complex fluid collection causing significant symptoms']
- S d - Chronic walled-off or large fluid collection causing life-threatening symptoms
  [instead of 'Large complex fluid collection causing life-threatening symptoms or a chronic walled-off collection']
